# Phylogenomics and Biogeography of *Populus* Based on Comprehensive Sampling Reveal Deep-Level Relationships and Multiple Intercontinental Dispersals

**DOI:** 10.3389/fpls.2022.813177

**Published:** 2022-02-04

**Authors:** Yachao Wang, Jin Huang, Enze Li, Shenjian Xu, Zhenfeng Zhan, Xuejiao Zhang, Zhiqi Yang, Feiyi Guo, Kangjia Liu, Dong Liu, Xueli Shen, Ce Shang, Zhixiang Zhang

**Affiliations:** Laboratory of Systematic Evolution and Biogeography of Woody Plants, School of Ecology and Nature Conservation, Beijing Forestry University, Beijing, China

**Keywords:** phylogenomics, biogeography, *Populus*, cytonuclear discordance, species complex, subgenus *Abaso*

## Abstract

*Populus* not only has significant economic and ecological values, but also serves as a model tree that is widely used in the basic research of tree growth, physiology, and genetics. However, high levels of morphological variation and extensive interspecific hybridization of *Populus* pose an obstacle for taxonomy, and also to the understanding of phylogenetic interspecific relationships and biogeographical history. In this study, a total of 103 accessions representing almost all the wild species of *Populus* were collected and whole-genome re-sequenced to examine the phylogenetic relationships and biogeography history. On the basis of 12,916,788 nuclear single nucleotide polymorphisms (SNPs), we reconstructed backbone phylogenies using concatenate and coalescent methods, we highly disentangled the species relationships of *Populus*, and several problematic taxa were treated as species complexes. Furthermore, the phylogeny of the chloroplast genome showed extensive discordance with the trees from the nuclear genome data, and due to extensive chloroplast capture and hybridization of *Populus* species, plastomes could not accurately evaluate interspecies relationships. Ancient gene flow between clades and some hybridization events were also identified by ABBA–BABA analysis. The reconstruction of chronogram and ancestral distributions suggested that North America was the original region of this genus, and subsequent long dispersal and migration across land bridges were contributed to the modern range of *Populus*. The diversification of *Populus* mainly occurred in East Asia in recent 15 Ma, possibly promoted by the uplift of the Tibetan Plateau. This study provided comprehensive evidence on the phylogeny of *Populus* and proposed a four-subgeneric classification and a new status, subgenus *Abaso*. Meanwhile, ancestral distribution reconstruction with nuclear data advanced the understanding of the biogeographic history of *Populus*.

## Introduction

*Populus*, collectively known as poplar, aspen, cottonwood, or “Yang Shu” in Chinese, is widely distributed from subtropical to boreal forests in the northern hemisphere, primarily in temperate forests (Ding, 1995; Eckenwalder, 1996). In native ecosystems, trees of the genus *Populus* play a major role, recolonizing sites after disturbances and providing important habitats for wildlife. Owing to its excellent features, poplars have been planted in many parts of the world and have become a highly promising crop option. They are not only widely used in windbreaks, protective stands to prevent soil erosion, and in row or gallery plantings to stabilize the banks of streams and canals, but also show impressive productivity for desirable wood products (Stettler et al., 1996). Furthermore, poplar is also recognized as an excellent model tree for the study of genetics, tree growth, and its underlying physiology for its fast growth rate, easy vegetative propagation, and relatively small genome (Stettler et al., 1996; Bradshaw et al., 2000; Cronk, 2005). The first completion of tree genome sequence of *P*opulus *trichocarpa* also shed new light on the exploration of wood development, nutrient and water movement, crown development, and disease resistance in trees (Tuskan et al., 2006). Moreover, a clear understanding of the evolutionary history of *Populus* will provide an important foundation for biological studies and genetic breeding programs.

Even though vastly valuable, the genus *Populus* has consistently been considered a troublesome taxonomic group. It is widely accepted that *Populus* consists of six sections [*Abaso*, *Turanga*, *Populus* (synonym: *Leuce*), *Leucoides*, *Aigeiros*, and *Tacamahaca*], primarily based on morphological evidence (Eckenwalder, 1996). However, there is still much debate over species delimitation and classification. Depending on classification predilections, approximately 22–100 natural species and hundreds of hybrids and cultivars have been proposed for this genus (Dickmann and Stuart, 1983; Eckenwalder, 1996; Fang et al., 1999; Chao et al., 2009; Slavov and Zhelev, 2012). Flora of China (Fang et al., 1999) recorded 71 species within *Populus* in China and reckoned that nearly 100 species exist throughout the world. On the other hand, Chinese taxonomists were criticized as “splitters,” who wish to give formal recognition to any entries that can be recognized in the field and herbaria and were reluctant to accept within species variation, instead of “lumpers,” who accept a broader range of variation within species (Eckenwalder, 1996). Thus, it is particularly important to detect the interspecies variation and phylogenetic relationships of *Populus* using genetic evidence.

Molecular methods have been used to analyze the phylogenetic relationships of *Populus*, but the results have not consistently supported morphological classifications, and intersectional relationships have been the subject of controversy. Three non-coding regions of chloroplast *trnT*-*trnF* and two ITS sequences were used to reconstruct the phylogeny of 17 species represented sect. *Tacamahaca*, sect. *Aigeiros*, and sect. *Populus*; the results showed that sections *Tacamahaca* and *Aigeiros* were polyphyletic (Hamzeh and Dayanandan, 2004). In another phylogenetic study, based on four chloroplast fragments (*rbcL*-*a*, *psbI*-*psbK*, *psbA*-*trnH*, and *trnL*-*trnF*), the monophyly of sections *Leucoides* and *Populus* were recovered, but sect. Tacamahaca were divided into two distinct clades (Yun et al., 2015). Moreover, a study based on 23 single-copy nuclear DNA and 34 chloroplast fragments firstly suggested the phylogenetic positions of the sixth section, sect. *Abaso* (only include *Populus mexicana*) (Liu X. et al., 2017). With the combination of nuclear and chloroplast DNA fragments, sect. *Tacamahaca* and sect. *Aigeiros* have usually been inferred to be polyphyletic or hybrid origin (Hamzeh and Dayanandan, 2004; Wang et al., 2014; Yun et al., 2015; Liu X. et al., 2017). Nevertheless, phylogenetic positions of many species have not been resolved owing to limited genetic information from molecular markers and DNA fragments. Chloroplast genomes can provide much more informative sites to reconstruct a more solid phylogeny which illustrated the relationship of maternal lineage. Five major clades have been recovered within *Populus* in previous plastome studies (Zhang L. et al., 2018; Zong et al., 2019). Sect. *Populus* formed a monophyletic clade with *Populus nigra* nested, species of sect. *Leucoides*, sect. *Tacamahaca*, and sect. *Aigeiros* expressed polyphyletic relationships. However, topology based on the whole length of genomes and result from 77 chloroplast protein-coding genes were inconsistent (Zhang L. et al., 2018; Zong et al., 2019). For *Populus*, chloroplast data seldom accurately reflect species relationships due to the severe effects of chloroplast capture (Liu X. et al., 2017) and hybridization. Whole-genomic data extracted from next-generation sequencing has been extensively used to resolve complicated systematic problems (Xu et al., 2012, 2014; Zhou et al., 2014; Chen et al., 2016, 2018). With the support of low coverage whole-genome sequencing, four main clades from 29 *Populus* species were recovered with high bootstrap supports (Wang et al., 2020) Sect. *Abaso*, sect. *Turanga*, and sect. *Populus* were all well-founded monophylies. The last clade, which was named ATL clade in Wang et al. (2020), consisted of three polyphyletic sections, sect. *Leucoides*, sect. *Aigeiros*, and sect. *Tacamahaca*. These three sections were ever morphologically identified as subg. *Eupopulus* (Dode, 1905). *Hitherto*, a relatively large number of *Eupopulus* species were not involved in previous phylogenetic studies, and molecular makers also should be improved to provide higher resolution. In addition, hybridization prevented the sole use of chloroplast genome to address these issues. Therefore, whole-genome data and comprehensive sampling should be conducted to decipher the phylogenetic relationships of *Populus*.

*Populus* has a widespread distribution throughout the northern hemisphere, and its diversification center and distribution center are in East Asia (Gong, 2004). However, the origin and diffusion of *Populus* still remain unclear and controversial. Two main hypotheses about the area of origin, China or North America, have been proposed. First, owing to the highest degree of diversification and the earliest fossils (*Populus latior*) in East Asia, *Populus* was considered to have originated from East Asia and spread from east to west (Ding, 1995; Gong, 2004). In contrast, another hypothesis is that the *Populus* species first appeared in North America and then dispersed to other continents, primarily because the earliest fossil records were found in North America (Liu X. et al., 2017). The earliest leaves fossils in North America that resembled *Populus* were also present in the Paleocene, and the first credible fossils with a rare twig bearing both leaves and fruits of *Populus* were identified in UT, United States and attributed to sect. *Abaso* (Collinson, 1992; Eckenwalder, 1996). Fossils of the extinct sister genus of *Populus* and *Salix*, *Pseudosalix*, were also found at the same formation (Boucher et al., 2003). The occurrence of them raised the possibility of a North American origin of Salicaceae. Alternatively, the phylogenetic position of *P. mexicana* provides additional evidence for this hypothesis. This only extant species of sect. *Abaso*, which is restricted to Mexico, was supported as the basal taxon of *Populus* phylogeny using nuclear markers (Liu X. et al., 2017; Wang et al., 2020). Both assumptions merely consulted morphological and fossil evidence, and no robust phylogenetic studies have been reconstructed to provide evidence for the origin and evolution of *Populus*.

Since previous phylogenetic examinations involved limited sequencing techniques or incomplete sampling, interspecific relationships of *Populus* still remain unclear. Thus, its divergence and biogeographical history were not yet well defined. A well-resolved phylogeny is the key to understand the evolutionary history and patterns of species diversification of *Populus*. In this study, with extensive and nearly complete sampling, we respectively reconstructed the backbone phylogeny for *Populus* based on variants identified from nuclear genomic data and complete chloroplast genomes to clarify the phylogenetic relationships of *Populus*, especially subg. *Eupopulus*. These two phylogenies were then compared to examine the conflicts between biparental and maternal inheritance. We further reconstructed the divergence and biogeographical history of *Populus*.

## Materials and Methods

### Plant Material

Sampling was conducted primarily following the taxonomy system of Eckenwalder (Eckenwalder, 1996) and the Flora of China (Fang et al., 1999). We also referred to other treatises and databases, including The Woody Plants of Korea^1^, Salicaceae of Japan (Hiroyoshi, 2001), the flora of USSR (Nazarov, 1936), Kenya trees, shrubs, and Lianas (Beentje et al., 1994), the Euro + Med PlantBase^2^, and The Plant List^3^. In total, 103 accessions of 54 *Populus* species were collected, which represented nearly all of the native species, as well as two variants and two hybrid species (Supplementary Table 1). The cultivated species and artificial hybrids were not included. Fresh leaves of 80 samples were collected from natural adult trees and dried using silica gel. The voucher herbarium specimens were deposited in the Museum of Beijing Forestry University (BJFC) and Herbarium, Institute of Botany, CAS (PE), Beijing, China. Three tissue samples were obtained from New York Botanical Garden DNA Bank (New York, NY, United States), and the images of voucher herbarium specimens were available on the web of the New York Botanical Garden^4^. Two tissue samples of *P. mexicana* were transferred from the Florida Museum of Natural History (Gainesville, FL, United States) and University of Arizona Campus Arboretum (Tucson, AZ, United States), respectively. The images of voucher herbarium specimens or live tree were also available on their website^5, 6^. Six DNA samples were obtained from the DNA and Tissue Bank at Kew^7^. In summary, most of taxa were sampled by 1–2 accessions. Considering the wide distribution or disputed classification, *P. nigra*, *Populus pseudoglauca*, *Populus szechuanica*, *Populus rockii*, *P. szechuanica* var. *tibetica*, *Populus cathayana*, and *Populus koreana* were collected from 3 to 5 accessions, respectively. In addition, previous sequencing data of 12 samples were included from Sequence Read Archive (SRA) in the National Center for Biotechnology Information (NCBI) database and the Genome Sequence Archive (GSA) in the BIG Data Center, Beijing Institute of Genomics (BIG), Chinese Academy of Sciences, respectively. Two *Salix* species were used as outgroups.

### Sequencing and Quality Control

We utilized the CTAB protocol with minor modifications to extract the whole-genomic DNA from the tissue samples (Doyle, 1987). All of the DNA samples were shipped to Novogene^8^ (China) for subsequent sequencing. After the DNA quality assessment, each sample was sheared to construct a pair-end sequencing library with an insert size of *c.* 350 bp and sequenced using an Illumina HiSeq 4000 platform (Illumina, San Diego, CA, United States).

Low-quality reads of raw sequencing data were removed with the following criteria: (1) the proportion of N was greater than 10%, and (2) the proportion of low-quality bases (Q ≤ 5) was greater than 50%. The clean data from sequencing company were than inspected and filtered by FastQC^9^ and trimmomatic v.0.36 (Bolger et al., 2014). The bases with a quality <3 at the head and tail were filtered. A sliding window of size 4 bp was used to filter the bases with a mean quality <15. At last, reads with a size not <50 bp were retained.

### Nuclear Variants Discovery

A BWA-SAMtools-GATK pipeline was performed to discover variants. First, Bwa-MEM v0.7.17-r1188 (Li and Durbin, 2009) with default parameters was used to map pair-end resequencing reads of each sample to the nuclear genome of *P. trichocarpa* (Tuskan et al., 2006). Secondly, mapping reads were converted to a BAM file and sorted using the SAMtools package v1.6 with the command “-bF 4 -q 20” filtered (Li et al., 2009). PCR duplication was marked using Picard tools v.2.1.1^10^. Short variants were called using the Genome Analysis Toolkit (GATK) v4.1.4 (McKenna et al., 2010) with HaplotypeCaller and GenotypeGVCFs tools. Hard filters implemented in GATK were applied to the raw variants with the parameters as “QD < 10.0 | | FS > 60.0 | | MQ < 40.0 | | MQRankSum <−12.5 | | ReadPosRankSum <−8.0,” and missing data was filtered using vcftools v 0.1.17 (Danecek et al., 2011). SNPs were extracted using the SelectVariants tool implemented in GATK. The final set was merged with outgroups set implemented in GATK v3.8.0 with the CombineVariants tool (McKenna et al., 2010). SNPs were annotated referring to the *P. trichocarpa* genome using SnpEff v4.3t^11^. A principal component analysis (PCA) of *Populus* was performed using plink v1.90 (Purcell et al., 2007) based on these SNPs of the whole genome.

### Complete Chloroplast Genomes Assembly

Geneious Primer (Kearse et al., 2012) was used to assemble the chloroplast genome. The paired-end reads were assembled to the reference chloroplast genome of *P. trichocarpa* (Tuskan et al., 2006). The Map tool with default parameters was used to filter the available reads, which were used for *de novo* assembling. Medium-low sensitivity was used. We then used the Fine Tuning function to bridge the gaps. Complete and partial assemblages for the organelle genomes were remapped using original reads. Plann (Huang and Cronk, 2015) was employed to annotate the chloroplast genome sequence, and all the annotations were manually corrected using the Geneious Primer.

### Phylogeny Inference

A maximum likelihood (ML) phylogenetic tree based on whole-genomic SNPs was constructed using IQ-TREE v1.6.9 (Nguyen et al., 2014). The best model was proposed by ModelFinder (Kalyaanamoorthy et al., 2017) implemented in IQ-TREE. The phylogeny was inferred simultaneously with 1,000 ultrafast bootstrap approximation (Hoang et al., 2018) and a 1,000 SH-aLRT branch test (Guindon et al., 2010).

We also conducted two coalescent approaches to reconstruct the species tree. The sliding-window method is the most common practice to build a species tree, and has been utilized in studies of bacteria, insects, mammals, and plants (Takuno et al., 2012; Zhang et al., 2016; Liu Y. et al., 2017; Chen et al., 2019). Thus, concatenated SNPs were filtered as 10-kb non-overlapping windows across the genomic alignments as “super-genes” for tree constructions. The gene trees were constructed using RAxML v8.2.11 (Stamatakis, 2014) based on the GTRGAMMA model. The fast bootstrap was conducted for 100 times with the option “-f a.” Finally, the species tree was inferred using ASTRAL v5.15.1 (Zhang C. et al., 2018). In addition to evaluating the gene tree discordance, an alternative quartet topologies test to show quartet supports for the tree topologies was conducted using the -t 8 option in ASTRAL. Additionally, we performed an SVDquartets analysis (Chifman and Kubatko, 2014) in PAUP* v4.0a168 (Swofford, 2003) using the concatenated SNPs with 100 bootstrap replicates and all quartets sampled to infer a species tree.

The plastome sequences with one inverted repeat (IR) removed were aligned using the MAFFT online version with the default parameter (Katoh et al., 2005). The ML phylogenetic tree was reconstructed using IQ-TREE v1.6.9 (Nguyen et al., 2014) based on chloroplast data. The parameter settings were the same as above.

### ABBA–BABA Analysis

At first, an alignment-based method was used to infer the ancestral state and the nuclear genome of *Salix dunnii* (He et al., 2021) was aligned to the *P. trichocarpa* reference genome (Tuskan et al., 2006) using minimap2 (Li, 2018). The alignment was transferred to variant call format (VCF) using SAMtools v1.6 (Li et al., 2009) and BCFtools v1.11 (Danecek and McCarthy, 2017). Then, the ABBA–BABA test was performed using Dsuite v0.3 (Malinsky et al., 2021) to assess evidence for introgression of *Populus*. The *Patterson*’s *D*-statistic (Durand et al., 2011) was calculated using the Dtrios program from Dsuite. In brief, for the ordered alignment {[(P1, P2), P3], O}, the ABBA site pattern refers the shared derived alleles of P2 and P3, and BABA site pattern refers to the shared derived alleles of P1 and P3. Under the null hypothesis of incomplete lineage sorting (ILS), the number of ABBA sites and BABA sites is expected to be equal (*D* = 0). Alternatively, significant deviation of *D* from 0 suggests other events, in particular P3 exchanging genes with P1 or P2 (Durand et al., 2011).

### Divergence Time Estimation

The best-preserved fossils of *Populus* with a rare twig that bore both leaves and fruits originate from the Eocene Green River Formation in Utah, Colorado, and Wyoming in the United States, and these fossils occurred in the early Middle Eocene (∼48 Ma) (Boucher et al., 2003; Manchester et al., 2006). All of them were similar to *P. mexicana* (Manchester et al., 1986) and attributed to sect. *Abas*o (Eckenwalder, 1977; Manchester et al., 2006). At the same formation, another famous fossil is *Pseudosalix handleyi*, which also has leaves and fruits attached and represents a lineage separate from *Populus* and indicates that *Populus* (and likely *Salix*) represented a unique lineage(s) at this time (Boucher et al., 2003). Thus, the divergence time between *Populus* and *Salix* was constrained to 48.13 and 48.22 Ma. Another relatively reliable fossil was recorded in the Middle Miocene, designated *Populus zhenyuanensis* (Liang et al., 2016), which is similar to extant *P. szechuanica* in leaf shape, size, and surface detail. Therefore, the divergence age between the *P. szechuanica* clade (including *P. szechuanica*, *P. rockii*, and *Populus xiangchengensis*) and the *Populus ciliata* clade (including *P. ciliata*, *Populus yatungensis*, *P. szechuanica* var. *tibetica*, *Populus haoana*, *P. pseudoglauca*, and *Populus mainlingensis*) was calibrated to 11.61–15.97 Ma.

Since the monophyly of many species was not recovered in the plastomes tree, which only represented the maternal lineage, nuclear genome SNPs data was used to determine the divergence times. With the exception of two hybrids, *Populus wenxianica* (presented as a cultispecies) and redundant accessions, a new set, including 55 *Populus* individuals and two outgroups, was generated to estimated divergence dates. Each species or variety remained as only one accession, but *P. szechuanica* and *P. koreana* remained as two. At first, a new tree was reconstructed as the input tree using IQ-TREE v1.6.9 (Nguyen et al., 2014) with the same parameter as above. Then, two distinct runs were conducted with the same approximation likelihood analysis using MCMCtree of the PAML package (Yang, 2007). The model of site substitute was set as GTR. A Markov Chain Monte Carlo (MCMC) run was discarded for the first 40,000,000 burn-in iterations. Sampling was taken at every 1,000 iterations with 100,000 times.

### Ancestral Area Reconstruction

To infer the ancestral geographic distributions, the R package “BioGeoBEARS” (Matzke, 2013) implemented in RASP (Yu et al., 2015) was used to test the best model. The DEC + J (Dispersal-Extinction-Cladogenesis) model with the highest AIC_wt value was selected to reconstruct the ancestral geographic distributions. The divergence tree reconstructed by MCMCtree was used as the input tree. According to the distribution of extant *Populus* species and extinct fossils, four operational geographic areas were divided into (A) Europe, North Africa, West-Central Asia and Xinjiang, China; (B) North Asia, East Asia and the edge of Himalayas; (C) Kenya; and (D) North America. The boundaries of the four areas were defined referring to the biogeographical regionalization of distributions of dicotyledonous plants in the world (Shen et al., 2018). In addition, the maximum number of areas was set to four.

## Results

### Sequence Data Processing

We resequenced 91 accessions, and 676.58 Gb raw data was generated. Totally, 735.14 Gb clean data of 103 individuals were obtained for subsequent analysis. After a series of strict analyses, an average of 56.71% reads was mapped to the genome *P. trichocarpa*. The average coverage depth and average mapping coverage of the genome *P. trichocarpa* were 10.93× and 78.20%, respectively (Supplementary Table 1). Finally, 12,916,788 high-quality SNPs were obtained (Supplementary Table 3).

In total, we assembled 103 complete chloroplast genomes, which had a common typical quadripartite structure, containing a large single-copy (LSC) region and a small single-copy (SSC) region separated by two IRs. The plastid genomes length of these 103 accessions ranged from 155,170 (*Populus grandidentata* #1) to 158,474 bp (*Populus ilicifolia* #1) (Supplementary Table 2). The plastomes of almost all the species have been identified with 129 genes, composing 85 coding sequences (CDSs), 8 rRNAs, and 36 tRNAs. Owing to the loss of two *rps7* genes, four species, *Populus pruinosa*, *P. pseudoglauca*, *P. mainlingensis*, and *Populus glauca*, contained only 127 genes, respectively (Supplementary Table 2).

### Phylogeny Based on Nuclear Single Nucleotide Polymorphisms

**FIGURE 1:**
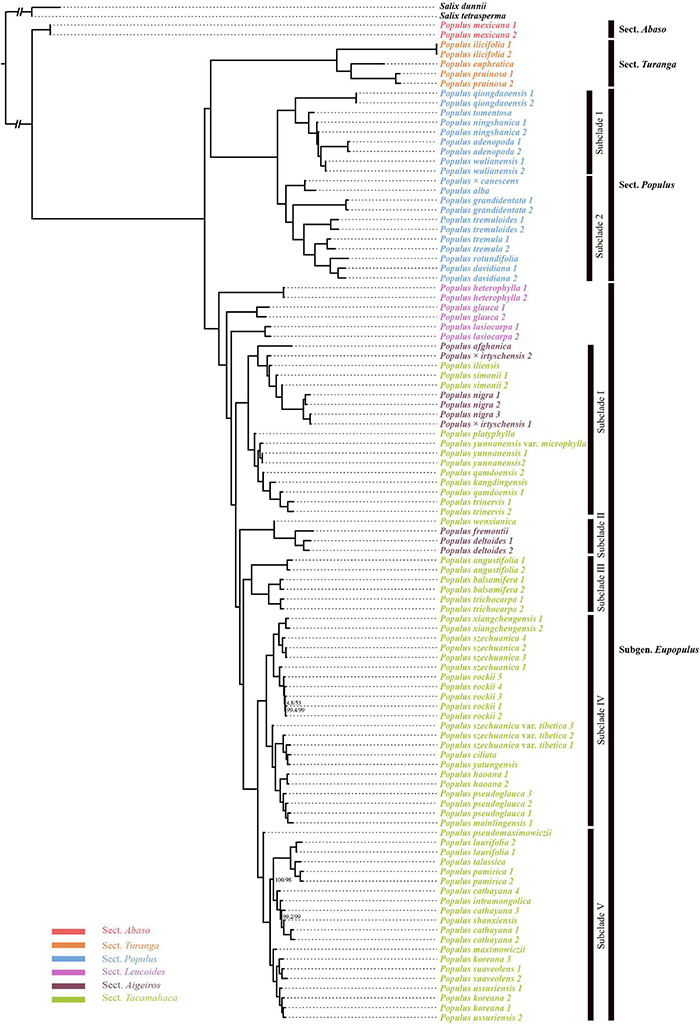
Phylogenetic relationship of the genus *Populus* reconstructed by IQ-TREE based on 12,916,788 nuclear SNPs. Unless otherwise indicated, all nodes had 100% supports of SH-aLRT bootstrap (Alrt) and Ultrafast bootstrap (UFBoot).

**FIGURE 2:**
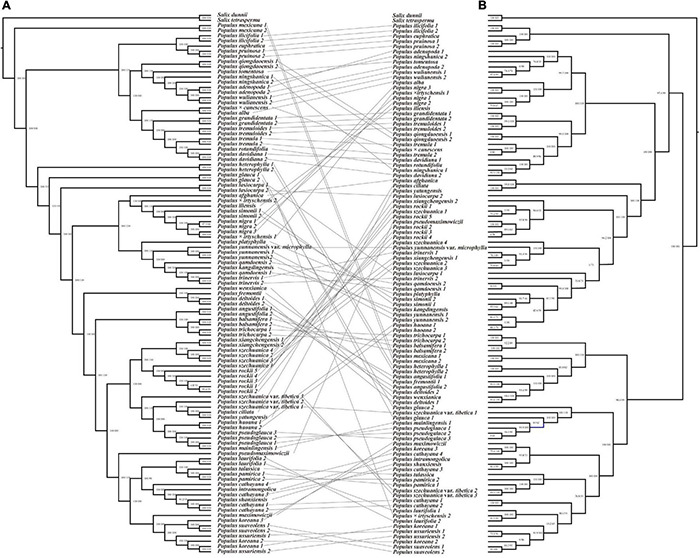
Cytonuclear conflicts among different *Populus* lineages. The taxa that encountered cytonuclear conflicts are linked by black lines. **(A)** Phylogenetic relationship of the genus *Populus* reconstructed by IQ-TREE based on 12,916,788 nuclear SNPs. **(B)** Phylogenetic relationship of the genus *Populus* reconstructed by IQ-TREE based on complete chloroplast genomes. The values indicate the supports of SH-aLRT bootstrap (Alrt) and Ultrafast bootstrap (UFBoot).

For the 12,916,788 SNPs data set, 3,110,298 were constant; 1,878,806 were singleton sites, and 7,927,684 were parsimony-informative sites. The ML tree recovered four main clades of *Populus*. *P. mexicana*, the only extant species of sect. *Abaso*, diverged first (Figure 1). Sect. *Turanga* was monophyletic and was the sister of another monophyletic clade, sect. *Populus*. Species of sect. *Leucoides*, sect. *Aigeiros*, and sect. *Tacamahaca* together formed a monophyletic clade, which was a sister to sect. *Turanga* + sect. *Populus* clade. These three sections were ever morphologically identified as subg. *Eupopulus* (Dode, 1905) and named ATL clade in Wang et al. (2020). Three species of sect. *Leucoides* diverged successively at the base of subg. *Eupopulus* (Figures 1, 2A). In addition, we also reconstructed a concatenate phylogeny using SNPs from Wang et al. (2020) with the same method as above and recovered the same topology (Supplementary Figure 2).

Five subclades were recovered within sect. *Aigeiros* + sect. *Tacamahaca*. Subclade I diverged first and contained seven species and one variety of sect. *Tacamahaca*, two species and a hybrid of sect. *Aigeiros*. Subclade II subsequently diverged and included two species of sect. *Aigeiros* and one species of sect. *Tacamahaca*. Subclade III included three species of sect. *Tacamahaca*. The remaining species of sect. *Tacamahaca* were divided into subclades IV and V. Seven species and one variety were contained in subclade IV, and 11 species composed of subclade V. The species of each subclade and its distribution are shown in Figure 1 and Supplementary Table 4.

**FIGURE 3:**
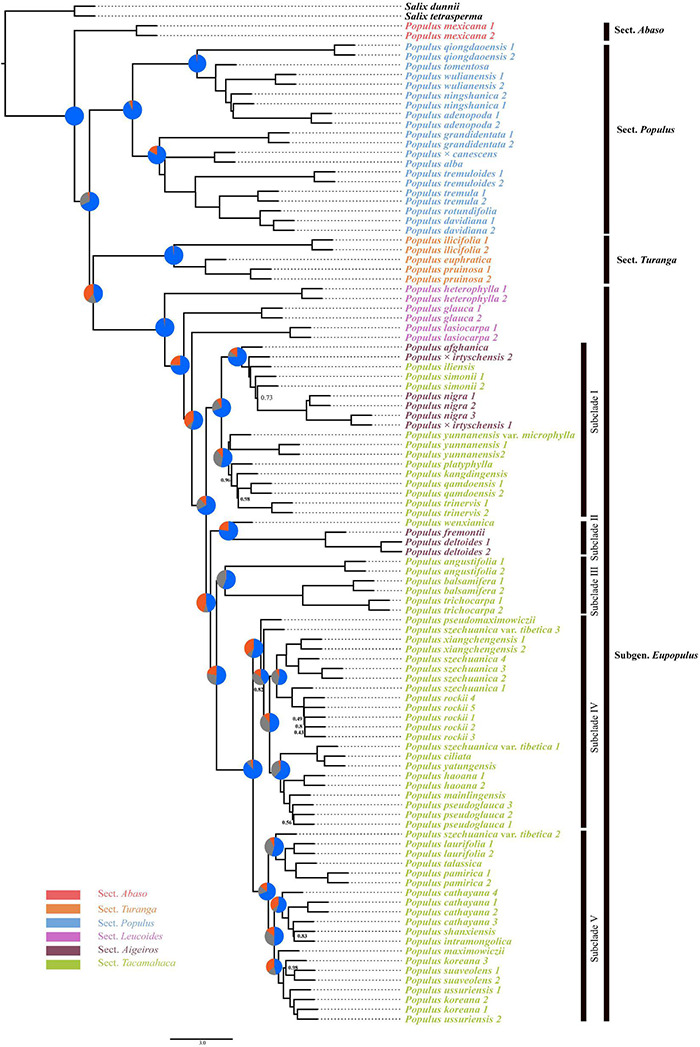
Phylogeny estimated with alternative quartet topologies (-t 8) in ASTRAL based on 10 kb non-overlapping windows. Unless otherwise indicated, all nodes had 1.0 posterior probability. Pie charts present the quartet support for the main topology (blue), the first alternative (gray), and the second alternative (orange).

The species trees inferred from two coalescent methods also supported the four main clades of *Populus* and similar five subclades within sect. *Aigeiros* + sect. *Tacamahaca*, but topologies conflicted with the concatenated genome tree and each other (Figure 3 and Supplementary Figure 1). The basal position of sect. *Abaso* was also recovered based on ASTAL method, but sect. *Populus* diverged earlier than sect. *Turanga* and subg. *Eupopulus* (Figure 3). The SVDquartets analysis revealed the first divergence of subg. *Eupopulus*, and successive sect. *Populus*, sect. *Abaso* and sect. *Turanga* (Supplementary Figure 1). Additionally, the phylogenetic position of some supposed hybrid taxa of subg. *Eupopulus* severely conflicted with the concatenated genome tree, such as *P. wenxianica*, *P. szechuanica* var. *tibetica*, and *Populus pseudomaximowiczii.*

### Phylogeny Based on Chloroplast Genomes

The plastome data matrix consisted of 174,083 characters, of which 166,358 were constant; 1,677 were singleton sites, and 6,048 were parsimony-informative sites. Compared with the nuclear genome tree, the phylogeny reconstructed by complete chloroplast genomes presented a quite different topology (Figure 2B and Supplementary Figure 3). The genus *Populus* was divided into five clades (Alrt > 95, UFB ≥ 99), and only sect. *Turanga* was monophyletic with full support (Clade A). Moreover, Sect. *Populus* clustered together as Clade B, with *P. nigra*, *Populus* × *irtyschensis #*1 of sect. *Aigeiros* and *Populus iliensis* of sect. *Tacamahaca* nested. Clade C was composed of *Populus afghanica* of sect. *Aigeiros*, *Populus lasiocarpa* of sect. *Leucoides* and 15 Asian species of sect. *Tacamahaca*. With the exception of two species of sect. *Populus*, *Populus tremuloides* and *P. grandidentata*, all of the other North American species formed a monophyletic clade, clade D, which was the sister to clade E. Clade E was composed of *P. glauca* of sect. *Leucoides* and the rest of Asian species of sect. *Tacamahaca* with *P.* × *irtyschensis #*2 of sect. *Aigeiros* embedded. Not only do the relationships between sections differ from morphological classifications and the nuclear SNPs tree, but many species did not recover as monophyly, particularly in clade C and E, such as *P. lasiocarpa* and *P. koreana*.

### Introgression Analysis

Totally, the ancestral state identified from *S. dunnii* (He et al., 2021) covered about 82.8% of the whole genome SNPs. Taking *S. dunnii* as the outgroup for all comparisons, the introgression analysis between clades showed that sect. *Abaso* was more closely related to subg. *Eupopulus* than to other species of sections *Turanga* and *Populus* (Supplementary Figure 5a), which was similar to the results of Wang et al. (2020). We also found that *P. nigra* was more closed related to *Populus alba* than to any other species of sect. *Populus* (Supplementary Figure 5b). Further ABBA–BABA analysis showed that the degree of introgression of *P. szechuanica* var. *tibetica*, and *P. pamirica* was higher than that of other species of subg. *Eupopulus* (except *P. ciliata*), while *P. pseudomaximowiczii* was more closely related to *P. koreana* than any other species of subg. *Eupopulus* (except *P. rockii*, Supplementary Figures 5c,d). These results suggested an extensive and frequently gene flow history of *Populus*. These complicated admixture histories were also verified by the pervasive discordance between the nuclear tree and chloroplast tree (Figure 2).

### Analysis of Molecular Dating

**FIGURE 4:**
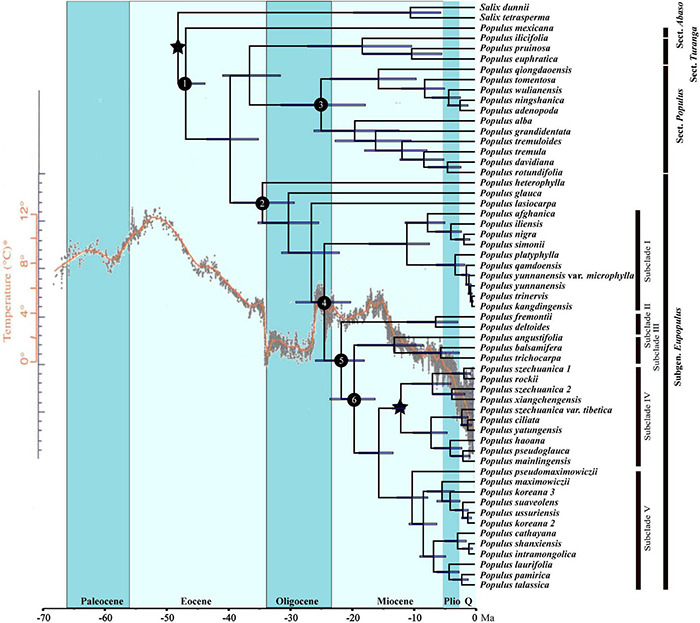
Chronogram of *Populus* inferred from the MCMCtree program in PAML. Blue bars indicate the 95% highest posterior density credibility intervals for node ages. Star indicates the calibration point. Nodes of interests were marked as 1–6. Q, quaternary; HPD, highest posterior density; Ma, million years ago. The phylogenetic chronogram used temperature curve as modified from Zachos et al. (2001) as background.

The divergence times s of genus *Populus* were estimated nearly identical by the two MCMCtree runs. The divergence age between *Populus* and *Salix* was constrained to 48.175 ± 0.045 Ma (million years ago) in the early Eocene. Section *Abaso* was estimated to diverge with other *Populus* species at 46.93 Ma [95% HPD (highest probability density): 43.80–48.14 Ma; node 1; Table 1 and Figure 4] in the middle Eocene, the next split of *Populus* species was at 39.72 Ma (95% HPD: 35.13–43.53 Ma; node 2; Table 1 and Figure 4). Two subclades of sect. *Populus* split at 24.97 Ma (95% HPD: 17.75–31.46 Ma; node 3; Table 1 and Figure 4) at the late Oligocene, almost at the same time at which subclade I of sect. *Aigeiros* + sect. *Tacamahaca* diverged from the other subclades (24.44 Ma, 95% HPD: 20.14–29.07 Ma; node 4; Table 1 and Figure 4). Diversification of the North American species of sect. *Aigeiros* + sect. *Tacamahaca* occurred at the early Miocene of 21.68 Ma (95% HPD: 17.91–25.87 Ma; node 5; Table 1 and Figure 4) and 19.58 Ma (95% HPD: 16.18–23.54 Ma; node 6; Table 1 and Figure 4), respectively.

**TABLE 1:** Estimated ages for the major nodes of *Populus*.

Node order	Mean age/Mya	95% highest posterior density interval (HPD)/Mya
1	46.93	43.80–48.14
2	39.72	35.13–43.53
3	24.97	17.75–31.46
4	24.44	20.14–29.07
5	21.68	17.91–25.87
6	19.58	16.18–23.54

### Ancestral Area Reconstruction

**FIGURE 5:**
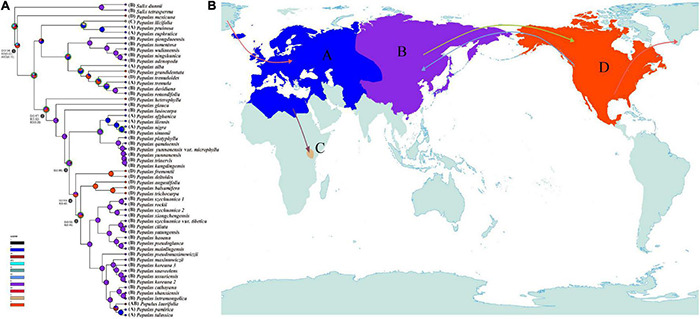
Ancestral areas of the genus *Populus*. **(A)** Ancestral area reconstructions of genus *Populus* using DEC + J model, implemented in RASP. Possible ancestral ranges and their respective probabilities are shown at each node. The four areas of endemism considered are as follows: (A) Europe, North Africa, West-Central Asia, and Xinjiang, China; (B) North Asia, East Asia, and the edge of Himalayas; (C) Kenya; and (D) North America. **(B)** Possible migration routes of the genus *Populus*. Solid arrows indicate inferred migration routes for the genus *Populus*.

The reconstruction of ancestral geographic distributions inferred that North America was the most probable original area of the genus *Populus* [D(0.34)/B(0.22)/BD(0.16), node 1; Figure 5A]. The ancestral area of subg. *Eupopulus* was also presumed to be North America [D(0.47)/B(0.32)/BD(0.20), node2; Figure 5A]. The common ancestral area of sect. *Aigeiros* + sect. *Tacamahaca* was reconstructed as North Asia, East Asia, and the edge of Himalayas [B(0.86), node 3; Figure 5A]. Moreover, 11 migration events were inferred with DEC + J model, and a pair of migration events from East Asia toward to North America and then spread back were also presumed to have occurred sequentially, as the common ancestral area subclade of III, IV, and V of sect. *Aigeiros* + sect. *Tacamahaca* was inferred to be North America [D(0.50)/B(0.45), node 5; Figure 5].

## Discussion

### Phylogenetic Relationships of *Populus*

With unprecedentedly complete sampling, the genus *Populus* was recovered as four clades with high supports based on nuclear SNPs (Figures 1, 3 and Supplementary Figure 1), while five clades were divided based on complete chloroplast genomes (Figure 2B and Supplementary Figure 3). Similar to the results of Zong et al. (2019), the complete plastome dataset provided high support for the divergence of five clades (Alrt > 95, UFB ≥ 99; Figure 2B and Supplementary Figure 3), but a previous study based on 77 chloroplast protein coding genes presented a different topology among the five clades and less supports in the second and the third clades (Zhang L. et al., 2018). Complete plastomes could more effectively resolve the phylogeny of poorly diverged *Populus* taxa compared with those diverged from protein coding gene sequences. Both biparental and maternal molecular phylogenies were contrasted with six sections acknowledged based on morphological characters (Eckenwalder, 1996). According to our results, the plastome phylogeny was highly inferior at solving interspecific relationships with few informative sites (5,720 parsimony–informative sites). In contrast, the nuclear genome could provide millions of variants (7,927,684 parsimony—informative sites) and be more effective at dissecting the deep phylogeny of *Populus*. In addition, different accessions of some species did not cluster with each other in the chloroplast genome tree, which reflected complex hybridizations. Therefore, the phylogenetic issues and the relationships among species primarily resulted from nuclear genomic phylogeny as follows.

With respect to nuclear genomic phylogeny, four clades were well supported, sect. *Abaso*, sect. *Turanga*, sect. *Populus*, and subg. *Eupopulus*. Sect. *Abaso*, the monotypic section distributed in the south of North America, was identified as a basal monotypic clade in most analysis, followed by the successive divergence of the other three clades: sect. *Turanga*, sect. *Populus*, and subg. *Eupopulus* (Figures 1, 2A, 3). SVDquartets analysis showed a quite different topology of these four clades. Wang et al. (2020) have recovered more topologies among these four clades and discovered more phylogenetic conflicts among different datasets or analysis methods. Especially, the internodes between these major clades were relatively short and each topology had low support values from gene trees [see Figures S4 and S5 from Wang et al. (2020) and Figure 3 in this study]. Furthermore, extensive gene flow among clades was also detected using ABBA–BABA and IBD (identity-by-descent) analysis (Wang et al., 2020). Species tree inference has also been presented to be inconsonant in the presence of gene flow (Solís-Lemus and Ané, 2016; Long and Kubatko, 2018). Thus, both gene flow and incomplete lineage sorting likely led to the phylogenetic inconsistence, and gene flow was likely to play the more important role (Wu, 1991; Wang et al., 2020).

Within subg. *Eupopulus*, there are no clear differences in the flowers and inflorescences between sect. *Tacamahaca* and sect. *Aigeiros*, and they have ever been considered to be accommodated in a single section (Eckenwalder, 1996). Alternatively, species of sect. *Tacamahaca* and sect. *Aigeiros* could be freely interfertile (Zsuffa, 1975; Eckenwalder, 1984), which primarily contributed to the conflict between the nuclear and chloroplast genome trees. These two sections can be divided into five subclades (Figures 1, 3). With more extensive sampling, more complex relationships were revealed within sect. *Aigeiros* + sect. *Tacamahaca* clade, even though the results were not entirely consistent with previous nuclear genome phylogeny described by Wang et al. (2020). For the subclade I, *Populus simonii* (sect. *Tacamahaca*) presented a close relationship with *P. nigra* (sect. *Aigeiros*); both of these species share the same characters of a flat petiole and narrowly ovoid and 2-valved capsules (Fang et al., 1999). However, Wang et al. (2020) showed that *Populus yunnanensis*, rather than *P. simonii*, was more closely related to *P. nigra*. In consideration of the fact that both *P. yunnanensis* and *P. simonii* have been cultivated for a long time, their origins are intricate and difficult to trace. Further studies based on more samples that utilize population genetic methods are needed to unravel the problem.

According to our results, some taxa were expected to be treated synonyms for another species. For instance, *P. mainlingensis* seemed to be a synonym of *P. pseudoglauca*, as they are monophyly in all the data sets (Figures 1–3). Alternatively, no significant morphological characters can distinguish these two species, and they are sympatrically distributed in southeast Tibet. Alternatively, a previous study also suggested that they are the same species (Chao, 1994). Likewise, both *Populus intramogolica* and *Populus shanxiensis* were highly similar to *P. cathayana* in morphology, although the previous two species were distinguished with the latter one based on the characters of leaf shape or pilosity, respectively (Wang and Tung, 1982; Sun, 1986). In our analysis, close relationships within these three species were recovered in both nuclear and chloroplast phylogenetic trees. Since the leaves of *P. cathayana* vary greatly, and the potential gene flow intensified the difficulty of resolving the relationship between *P. cathayana* and related species, *P. intromogolica* and *P. shanxiensis* seem to be synonyms of *P. cathayana*. Of course, deep study based on specimens and population genetics are needed to verify this hypothesis.

Additionally, some hybrid taxa could be detected by our concatenated and coalescent phylogenies. *P. wenxianica* was endemically distributed in Wenxian County, Gansu Province, China, rather than North America. Since a number of hybrid strains from *Populus deltoides* were cultivated in China for a long time, and the characters of the shape of leaf blades and the ovary carpel number differed between *P. wenxianica* and the two sect. *Aigeriros* species (Fang et al., 1999), we hypothesized that *P. wenxianica* was a hybrid from *P. deltoides* and another unknown parental ancestor. The leaf and capsule morphologies of *P. pseudomaximowiczii* were similar to those of *P. rockii*, but their distribution was discontinuous and cut off by the Taihang Mountains (Fang et al., 1999). But *P. koreana* is sympatric with *P. pseudomaximowiczii* in the Wuling Mountain, which increased the likelihood that hybridization took place. Given the conflict for the phylogenetic position of *P. pseudomaximowiczii* between concatenate and coalescent phylogenies, we hypothesized that *P. pseudomaximowiczii* is a hybrid taxon, and *P. rockii* could be the female parent, as supported by the chloroplast tree and ABBA–BABA test (Supplementary Figure 5c), and *P. koreana* could be another parent. Significantly, *P. szechuanica* var. *tibetica* did not cluster with *P. szechuanica*. We collected two individuals cultivated at Lhasa City and a native tree from Cuona County, Xizang Autonomous Region, China. They were found to be close to the Himalaya lineage in both biparental and maternal phylogenies, but the two cultivated samples were close to *P. pamirica* with respect to the chloroplast genome tree (Figure 2). *P. szechuanica* var. *tibetica* was first published with the specimen collected from a cultivated tree in Ladakh, and described as having smooth bark and nearly glabrous leaves (Schneider, 1916). *P. szechuanica* var. *tibetica* was once considered to be a variety of *P. pamirica* (Zhao and Liu, 2001) or a synonym of *P. ciliata* (Skvortsov, 2009). The conflicts among the nuclear concatenated, coalescent, and chloroplast trees and ABBA–BABA test demonstrated not only close relationship between *P. szechuanica* var. *tibetica* and *P. ciliata*, but potential hybridization between *P. szechuanica* var. *tibetica* and *P. pamirica* (Figure 2 and Supplementary Figure 5d).

Furthermore, the phylogenetic relationships of some species were still questionable and were treated as species complexes. *P. szechuanica #*1, collected from the type locality, was a sister to *P. rockii*, whereas *P. szechuanica #*2, *#*3, and *#*4 presented a close relationship with *P. xiangchengensis*, which was parapatric with *P. szechuanica* (Figures 1, 2A). These three taxa could be distinguished based on their angle branchlets, pilosity of branchlets, petiole, leaf veins, catkin rachis, and capsule (Fang et al., 1999). However, they were confused in the sympatric area. Thus, we considered these three species to be the *P. szechuanica* complex. The relationships within *Populus suaveolens* lineage were also confused and conflicted with morphological taxonomy, not only in the nuclear SNPs tree but also in the plastomes tree (Figure 2). It may be caused by extensive gene flow and perplexing classification within these four sympatric species. Further study should be conducted using more samples based on population genetics to further elucidate the relationships within these species complex.

### Cytonuclear Discordance in *Populus*

We reconstructed backbone phylogenetic trees based on nuclear genomic SNPs and complete chloroplast genomes, separately. They presented considerable conflicts and relatively short internodes between major clades (Figure 2). The incongruence between biparental and matrilineal phylogenetic trees commonly contributes to incomplete lineage sorting (ILS), convergent evolution or introgression (Degnan and Rosenberg, 2009; Cristina Acosta and Premoli, 2010; Pelser et al., 2010). There into, lineage sorting would not be expected to exhibit the strong geographical partitioning observed at the chloroplast genome tree (Figure 2B; Liu X. et al., 2017). Thus, cytonuclear conflicts of *Populus* were primarily caused by hybridization, or specifically, chloroplast capture and gene flow (recent hybridization).

Chloroplast capture, which introgressed a chloroplast genome from one plant or ongoing backcrossing of F1s with the parental populations, has frequently been thought to explain the inconsistencies between nuclear gene trees and cytoplasmic trees (Rieseberg and Soltis, 1991; Tsitrone et al., 2003; Cristina Acosta and Premoli, 2010). Sect. *Abaso* (*P. mexicana*) formed the basal clade in nuclear phylogeny but was close to *Populus heterophylla* in plastome phylogeny (Figure 2). Significant gene flow among them have been identified using ABBA–BABA tests (Wang et al., 2020). However, no IBD blocks were shared between these two clades, which implied an early gene flow event, and these IBD blocks were expunged by subsequent recombination. All this evidence pointed to a common hypothetical scenario that *P. mexicana* had suffered a significant chloroplast capture from the *P. heterophylla*-like ancestor (Liu X. et al., 2017; Wang et al., 2020). The high level of cytonuclear conflict also occurred in *P. nigra*, as *P. nigra*, *P. iliensis*, and *P.* × *irtyschensis #*1 nested among the members of section *Populus* in clade B of plastome tree (Figure 2B). Previous studies based on chloroplast genome data and morphological traits proposed a hypothesis that *P. nigra* was an introgression of the *P. alba* lineage and another unknown paternal lineage (Smith and Sytsma, 1990; Hamzeh and Dayanandan, 2004; Zong et al., 2019). Our ABBA–BABA analysis supported this scenario (Supplementary Figure 5b). Cytonuclear conflicts between deep clades were also likely caused by ancient chloroplast genome capture. Because sect. *Turanga* and sect. *Populus* maintained similar topologies in two phylogenies, the predecessors of these two sections should not have suffered chloroplast genome capture events. As shown in Figure 2, cytonuclear conflicts primarily occurred in subg. *Eupopulus*. With the exception of *P. nigra*, *P. iliensis* and one sample of *P.* × *irtyschensis*, subg. *Eupopulus* was divided into three clades in the chloroplast genome tree, clade C, D, and E (Supplementary Figure 3). As described above, the ancient chloroplast of these three exceptive taxa was captured from *P. alba* of sect. *Populus*. In the plastome tree, clade D, and E formed a monophyly and was a sister to the ancestors of sect. *Turanga* and sect. *Populus*, while clade C was a sister to sect. *Populus*. This topology suggested that the ancestors of clade C might suffered a chloroplast genome capture event from the ancestors of sect. *Populus*.

Alternatively, the interspecific gene flow is an evolutionary process that is responsible for generating gene tree discordance and therefore, hindering the estimation of species tree (Leaché et al., 2014). Recent hybridization can cause gene tree conflicts not only between species but also within species. IBD and ABBA–BABA analyses detected obvious gene flow between species within each clade in a previous study (Wang et al., 2020). In addition, species within sect. *Aigeiros* + sect. *Tacamahaca* that have overlapping ranges in China and North America are sexually compatible and hybridize freely (Eckenwalder, 1984; Mona et al., 2006; Schroeder and Fladung, 2010). The maternal phylogenetic relationships of intraspecies and interspecies of these two sections still remained puzzling, with different accessions of the same species not clustering together (Figure 2B). In addition, the topology of these species remained exceedingly incompatible within subg. *Eupopulus* (Figure 2). For example, two samples of *Populus trinervis* were found to be separated from each other in the chloroplast genome tree (Figure 2B), which suggested its maternal origin (chloroplast) would come from at least two maternal lineages. Obvious hybridization has also been found and indicates that *P.* × *irtyschensis* was the F1-dominent hybrid between *P. nigra* and *Populus laurifolia* (Jiang et al., 2016). Our chloroplast genome tree also supported this hybridization event (Figure 2B and Supplementary Figure 3).

### Biogeographical History

Ancestral area reconstruction with DEC + J model based on nuclear SNPs phylogeny indicated that *Populus* originated in the New World (Figure 5). Both morphological and fossil evidence also support the hypothesis of North American origin (Manchester et al., 1986, 2006; Collinson, 1992; Eckenwalder, 1996). The first confirmed fossil of *Populus* in the early Middle Eocene, were attributed to sect. *Abaso* and occurred in North America (Collinson, 1992; Eckenwalder, 1996; Manchester et al., 2006). Though earlier leaves fossils of *Populus* were reported both in East Asia and North America, the fossils of these leaves could not be entirely identified as *Populus* (Guo, 1983; Collinson, 1992; Manchester et al., 2006). In addition, *P. mexicana*, the only extent species of sect. *Abaso*, which recovered as the base clade in species tree (Figures 1, 3), was also endemic in North America.

Land bridges also played a major role in the dispersal of *Populus*. The ancestors of sect. *Populus*, sect. *Turanga*, and subg. *Eupopulus* were presumed to have arrived in the Old World through the North Atlantic Land Bridge (NALB) and rapidly diverged within 5 Ma in the late Eocene (Figures 4, 5). After arriving the Old World, there has been a single migration from Eurasia to Africa that occurred in *P. ilicifolia* of sect. *Turanga*. The persistent warm and humid climate in the Eocene (McInerney and Wing, 2011) may have promoted the spread and the divergence of *Populus*. A dramatic decrease in temperature with 17 million years of cooling occurred at the Eocene–Oligocene boundary (∼33.7 Ma) (Zachos et al., 2001), which may have been the cause of extinction of many species of sect. *Abaso* and sect. *Leucoides* (Collinson, 1992; Budantsev, 2005). The extant *P. mexicana* that survived in Mexico and three sect. *Leucoides* species discontinuously distributed in southwest China and southeast America could have caused by the temperature drop and subsequent climate fluctuation. This persistent cooling and increasing aridification driven by the Eocene–Oligocene transition (Zachos and Kump, 2005; Dupont-Nivet et al., 2007; Abels et al., 2011; Passchier et al., 2013; Kalyaanamoorthy et al., 2017) seems to have led to the divergence between *P. heterophylla* and other species of subg. *Eupopulus* (Figures 4, 5). moreover, an interesting dispersal from East Asia to North America and a subsequent back and forth spread occurred from the late Oligocene to the early Miocene (node 3, 4, and 5; Figure 5). During this period, the Bering Land Bridge (BLB) was certainly available as a route (Tiffney, 2000; Tiffney and Manchester, 2001; Milne and Abbott, 2002). Another curiously intercontinental diffusion of sect. *Populus* occurred during the Miocene, a date range too young for the NALB yet very consistent with vicariance across the BLB, possibly as a result of climate cooling in Beringia before (Wolfe, 1980; Tiffney and Manchester, 2001; Gladenkov et al., 2002).

Though the main clades split early, most divergence of *Populus* occurred after 15 Ma (Figure 4), and mainly in East Asia. Southwest China also retained the most abundant *Populus* species. Similarly, most of the related species with distribution centers in the Tibetan plateau diverged after recent ∼15 Ma (Qiu et al., 2011). The uplift of the Himalayan-Tibetan plateau with significant geographical effects (Harris, 2006; Riesselman et al., 2007; Su et al., 2019) and drove the occurrence of new niches. The changes in climate and terrestrial ecosystems led to the retreat of a number of plants and provided strong opportunity for pioneers.

## Taxonomic Treatment

Phylogenetic results from this and an earlier study (Wang et al., 2020) clearly indicated four clades among species among six sections. The paraphyletic three sections (i.e., sects. *Aigeiros*, *Leucoides*, and *Tacamahaca*) could be resolved by sinking all these taxa into a single subgenus, “*Eupopulus*,” which was proposed by Dode (1905) for their resinous winter buds. As all the four clades are well-supported by both morphological and molecular evidence, and diverged prior to ca. 35 Ma, we here propose a new subgenus of *Populus*, and try to establish a four-subgeneric classification.

***Populus*** L., Sp. Pl. 1034. 1753, nom. cons. – Type: *Populus alba* L. (designated by Britton & A.Br., Ill. Fl. N. U. S. ed. 2. 1: 587. 1913).

*Diagnosis*. – Trees dioecious; bark smooth, rough or furrowed; monopodial or sympodial branching; pith often 5-angled at cross section; buds with several unequal scales, resinous or not. Leaves alternate, deciduous; petiole terete or flattened, leaf blade linear to rhombic ovate, dentate or serrate; often dimorphic on long and short shoots. Inflorescences axillary or terminal, catkins, pendulous; bracts apically toothed or laciniate, membranous, caducous; disc cupular or saucer-shaped; stamens 4-many, filaments free; anthers longitudinally dehiscent; ovary 2–4(5)-carpellate, 1-loculed, stigmas 2–4. Capsule 2–4 (or 5)-valved, ovoid or spherical. Seeds few to numerous, minute, with a long dense tuft of silky hairs.

*Distribution* & *Diversity*. – Ca. 40–50 species; worldwide, mostly in northern hemisphere: Asia, Europe, North America, and Africa (Kenya).

### Key to Subgenera of *Populus*

1. Anthers elongate, apiculate at apex; leaves variably shaped, usually linear from juvenile branchlet, and get broader when mature; disc of female flowers caducous.………………2
1. Anthers short and broad, truncate or emarginate at apex; leaves usually broadly ovate; disc of female flowers persistent.…………………………………………3
2. Growth sympodial, without terminal buds; mature leaves serrated at margin; carpels usually 2, rarely 3. ………………………………………subg. ***Abaso***
2. Growth monopodial; mature leaves with few coarse teeth; carpels usually 3, rarely 2. ………………subg. ***Turanga***
3. Winter buds very viscid; leaf margin serrated; bracts fringed but not ciliate; carpels 2–4(5). ……………………………subg. ***Eupopulus***
3. Winter buds tomentose but not resinous; leaf margin lobed, incised, or with sinuous teeth; bracts with long, straight marginal hairs; carpels 2. ……………………………………subg. ***Populus***
1. ***Populus*** subg. ***Abaso*** (Eckenw.) C. Shang, Y.C. Wang & Z.X. Zhang, ***stat. nov.***
Basionym: *Populus* sect. *Abaso* Eckenw. in J. Arnold Arbor. 58(3): 194. 1977 – Type: *Populus mexicana* Wesm. ex DC. Prodr. 16(2): 328. 1968.
*Diagnosis*. – Winter buds slightly viscid; petiole subterete; leaves of juvenile plants linear, leaves of adults broader; bracts linear, laciniate at the apex; disc cup-shaped, membranous, laciniate, deciduous after flowering; capsules 2-(seldom 3-) valved.
*Distribution & Diversity*. – Only 1 species, *Populus mexicana*, endemic to North America.
2. ***Populus*** subg. ***Turanga*** (Bunge) Dode, Bull. Soc. Hist. Nat. Autun 18: 172. 1905 ≡ *Populus* sect. *Turanga* Bunge in Mém. Acad. Imp. Sci. St.-Pétersbourg 7: 498. 1851 – Type: *Populus diversifolia* Schrenk (= *Populus euphratica* Oliver).
*Diagnosis*. – Branches sympodial, without terminal buds; buds not resinous; petiole subterete; leaves variably shaped, margin entire or with few teeth; bracts spatulate, membranous; disc membranous, lobed or parted, with sharp teeth, caducous; anthers long, apex apiculate; ovary long ovoid; capsules elongate, (2 or) 3-valved, stipitate.
*Distribution* & *Diversity*. – 3 species, central and west Asia, north Africa, and Kenya.
3. ***Populus*** subg. ***Eupopulus*** Dode, Bull. Soc. Hist. Nat. Autun 18: 192. 1905 – Type: *Populus balsamifera* L. (designated here).
= *Populus* subg. *Leucoides* (Spach) N. Chao & J. Liu in J. Sichuan For. Sci. 19 (4): 12. 1998; N. Chao et al. in J. Wuhan Bot. Res. 27 (1): 26. 2009.
*Diagnosis*. – Branches monopodial; buds very viscid, with a strongly balsamic odor; petiole terete or flattened, often sulcate; bracts not fringed; stamens 10–60; anthers long elliptic to globose; disc usually entire, persistent; style short or absent; stigma 2–4-lobed; capsules 2–4 (or 5)-valved.
*Distribution* & *Diversity*. – 25–35 species, Asia, Europe, and North America.
4. ***Populus*** subg. ***Populus*** – Type: *Populus alba* L. (designated by Green, Prop. Brit. Bot.: 192. 1929).
= *Populus* subg. *Lecue* (Duby) Dode, Bull. Soc. Hist. Nat. Autun 18: 176. 1905
*Diagnosis*. – Buds tomentose or glabrous; petioles flattened or subterete; leaf blade lobed, incised, or with sinuous teeth, abaxially usually tomentose when young; bracts laciniate, long ciliate; disc sinuate. Stamens 6–12(–20); stigma 2–4-lobed; capsules long ellipsoid or long ovoid, usually 2-valved.

*Distribution* & *Diversity*. – Ca. 10 species, Asia, Europe, and North America.

## Conclusion

In this study, we collected almost all the wild species of *Populus* and reconstructed robust phylogenies from nuclear genome and chloroplast genome data. The deep phylogenetic relationships of *Populus* species were well resolved based on nuclear genomic phylogeny. According to the phylogeny, a new status, subg. *Abaso* was proposed, and a new classification system of four subgenera was established. Conflicts among clades and the inconsistency of phylogenetic positions of some hybrid species were also detected by concatenated and coalescent methods. In contrast, chloroplast genomic phylogeny was composed of five clades and inadequately settled the relationships among species of *Populus*. The cytonuclear discordance within *Populus* was extensive primarily owing to chloroplast capture and gene flow and was supported by the ABBA–BABA analysis. A New World origin of *Populus* and several migration events through land bridges were suggested by the phylogeny, divergence time analyses, and biogeographic implications. Based on comprehensive sampling, this study inferred the clear evolutionary history of *Populus*, and some confused taxa were proposed as species complexes, such as *P. szechuanica* complex and *P. suaveolens* complex. Further study based on population genetics method and more samples should be conducted to clarify relationships of close species.

## Data Availability Statement

The original contributions presented in the study are publicly available. This data can be found here: All newly assembled plastomes were deposited in GeneBank with accession numbers MW376757 – MW376858 and MK288023. Resequencing raw read data are stored at the NCBI Sequence Read Archive (SRA) in the Bioproject PRJNA687326.

## Author Contributions

YW, CS, and ZXZ conceived the idea. YW, JH, ZFZ, XZ, ZY, and CS collected the plant materials. YW, EL, FG, KL, DL, and XS analyzed the data. YW wrote and revised the manuscript. CS and ZZ revised the manuscript. All authors contributed to the article and approved the submitted version.

## Conflict of Interest

The authors declare that the research was conducted in the absence of any commercial or financial relationships that could be construed as a potential conflict of interest.

## Publisher’s Note

All claims expressed in this article are solely those of the authors and do not necessarily represent those of their affiliated organizations, or those of the publisher, the editors and the reviewers. Any product that may be evaluated in this article, or claim that may be made by its manufacturer, is not guaranteed or endorsed by the publisher.

## Footnotes

1
https://florakorea.myspecies.info/en

2
http://ww2.bgbm.org/EuroPlusMed/

3
http://www.theplantlist.org/

4
https://www.nybg.org/

5
https://www.floridamuseum.ufl.edu/

6
https://apps.cals.arizona.edu/

7
https://dnabank.science.kew.org/

8
http://www.novogene.com

9
www.bioinformatics.babraham.ac.uk/projects/fastqc/

10
http://picard.sourceforge.net/

11
http://snpeff.sourceforge.net/

## References

[B1] Abels H. A. Dupont-Nivet G. Xiao G. Bosboom R. Krijgsman W. (2011). Step-wise change of Asian interior climate preceding the eocene–oligocene transition (EOT). *Palaeogeogr. Palaeoclimatol. Palaeoecol.* 299 399–412. 10.1016/j.palaeo.2010.11.028

[B2] Beentje H. Adamson J. Bhanderi D. (1994). *Kenya Trees, Shrubs, and Lianas.* Nairobi: National Museums of Kenya.

[B3] Bolger A. M. Lohse M. Usadel B. (2014). Trimmomatic: a flexible trimmer for Illumina sequence data. *Bioinformatics* 30 2114–2120. 10.1093/bioinformatics/btu170 24695404 PMC4103590

[B4] Boucher L. D. Manchester S. R. Judd W. S. (2003). An extinct genus of Salicaceae based on twigs with attached flowers, fruits, and foliage from the eocene green river formation of Utah and Colorado, USA. *Am. J. Bot.* 90 1389–1399. 10.3732/ajb.90.9.1389 21659238

[B5] Bradshaw H. D. Ceulemans R. Davis J. Stettler R. (2000). Emerging model systems in plant biology: poplar (*Populus*) as a model forest tree. *J. Plant Growth Regul.* 19 306–313. 10.1007/s003440000030

[B6] Budantsev L. (2005). *Fossil Flowering plants of Russia and Adjacent Countries (in Russian).* St. Petersburg: Russian Academy of Sciences Komarov Botanical Institute.

[B7] Chao N. (1994). Taxonomic study on Salicaceae in Sichuan and its adjacent regins (III) (in Chinese). *Sichuan For. Sci. Technol.* 15 1–11. 10.2307/2666489

[B8] Chao N. Liu J. Gong G. (2009). On the classification and distribution of the subfamily Populoideae (Salicaceae) (in Chinese). *J. Wuhan Bot. Res.* 27 23–40.

[B9] Chen C. Liu Z. Pan Q. Chen X. Wang H. Guo H. (2016). Genomic analyses reveal demographic history and temperate adaptation of the newly discovered honey bee subspecies *Apis mellifera sinisxinyuan* n. ssp. *Mol. Biol. Evol.* 33 1337–1348. 10.1093/molbev/msw017 26823447 PMC4839221

[B10] Chen L. Qiu Q. Jiang Y. Wang K. Lin Z. Li Z. (2019). Large-scale ruminant genome sequencing provides insights into their evolution and distinct traits. *Science* 364:eaav6202. 10.1126/science.aav6202 31221828

[B11] Chen N. Cai Y. Chen Q. Li R. Wang K. Huang Y. (2018). Whole-genome resequencing reveals world-wide ancestry and adaptive introgression events of domesticated cattle in East Asia. *Nat. Commun.* 9:2337. 10.1038/s41467-018-04737-0 29904051 PMC6002414

[B12] Chifman J. Kubatko L. (2014). Quartet inference from SNP data under the coalescent model. *Bioinformatics* 30 3317–3324. 10.1093/bioinformatics/btu530 25104814 PMC4296144

[B13] Collinson M. E. (1992). The early fossil history of Salicaceae: a brief review. *Proc. R. Soc. Edinb. B Biol. Sci.* 98 155–167. 10.1017/s0269727000007521

[B14] Cristina Acosta M. Premoli A. C. (2010). Evidence of chloroplast capture in South American *Nothofagus* (subgenus *Nothofagus*, Nothofagaceae). *Mol. Phylogenet. Evol.* 54 235–242. 10.1016/j.ympev.2009.08.008 19683588

[B15] Cronk Q. (2005). Plant eco-devo: the potential of poplar as a model organism: research review. *New Phytol.* 166 39–48. 10.1111/j.1469-8137.2005.01369.x 15760349

[B16] Danecek P. McCarthy S. A. (2017). BCFtools/csq: haplotype-aware variant consequences. *Bioinformatics* 33 2037–2039. 10.1093/bioinformatics/btx100 28205675 PMC5870570

[B17] Danecek P. Auton A. Abecasis G. Albers C. A. Banks E. DePristo M. A. (2011). The variant call format and VCFtools. *Bioinformatics* 27 2156–2158. 10.1093/bioinformatics/btr330 21653522 PMC3137218

[B18] Degnan J. H. Rosenberg N. A. (2009). Gene tree discordance, phylogenetic inference and the multispecies coalescent. *Trends Ecol. Evol.* 24 332–340. 10.1016/j.tree.2009.01.009 19307040

[B19] Dickmann D. I. Stuart K. (1983). The culture of poplars in Eastern North America. *Rhodora* 19 10–19. 10.1515/9780691221373-003

[B20] Ding T.-Y. (1995). Origin, divergence and geographical distribution of Salicaceae (in Chinese). *Acta Bot. Yunnanica* 17 277–290.

[B21] Dode L. A. (1905). Extraits d’une monographie inédite du genre *Populus*. *Bull. Soc. Hist. Nat. Autun.* 18 161–231.

[B22] Doyle J. J. (1987). A rapid DNA isolation procedure for small quantities of fresh leaf tissue. *Phytochem. Bull.* 19 11–15.

[B23] Dupont-Nivet G. Krijgsman W. Langereis C. G. Abels H. A. Dai S. Fang X. (2007). Tibetan plateau aridification linked to global cooling at the eocene–oligocene transition. *Nature* 445 635–638. 10.1038/nature05516 17287807

[B24] Durand E. Y. Patterson N. Reich D. Slatkin M. (2011). Testing for ancient admixture between closely related populations. *Mol. Biol. Evol.* 28 2239–2252. 10.1093/molbev/msr048 21325092 PMC3144383

[B25] Eckenwalder J. E. (1977). North American cottonwoods (*Populus*, Salicaceae) of sections *Abaso* and *Aigeiros*. *J. Arnold Arboretum.* 58 193–208.

[B26] Eckenwalder J. E. (1984). Natural intersectional hybridization between North American species of *Populus* (Salicaceae) in sections *Aigeiros* and *Tacamahaca*. II. Taxonomy. *Can. J. Bot.* 62 325–335. 10.1139/b84-051

[B27] Eckenwalder J. E. (1996). “Systematics and evolution of *Populus*,” in *Biology of Populus and Its Implications for Management and Conservation*, eds Stettler R. F. Bradshaw H. D. <suffix>Jr.</suffix>, Heilman P. E. Hinckley T. M. (Ottawa, ON: NRC Research Press).

[B28] Fang Z. F. Zhao S. D. Skvortsov A. K. (1999). “Populus,” in *Flora of China*, eds Wu Z. Y. Raven P. H. (Beijing: Missouri Botanical Garden).

[B29] Gladenkov A. Y. Oleinik A. E. Marincovich L. Barinov K. B. (2002). A refined age for the earliest opening of Bering Strait. *Palaeogeogr. Palaeoclimatol. Palaeoecol.* 183 321–328. 10.1016/S0031-0182(02)00249-3

[B30] Gong G. (2004). The geographic distribution and origin of populus L. (in Chinese). *J. Sichuan. Forestry. Sci. Technol.* 25, 25–30.

[B31] Guindon S. Dufayard J. F. Lefort V. Anisimova M. Hordijk W. Gascuel O. (2010). New algorithms and methods to estimate maximum-likelihood phylogenies: assessing the performance of PhyML 3.0. *Syst. Biol.* 59 307–321. 10.1093/sysbio/syq010 20525638

[B32] Guo S. (1983). *Discussion on Late Cretaceous and Tertiary Phytogeographic Regions and Ecological Environment in China.* Beijing: The Science Press.

[B33] Hamzeh M. Dayanandan S. (2004). Phylogeny of *Populus* (Salicaceae) based on nucleotide sequences of chloroplast trnT-trnF region and nuclear rDNA. *Am. J. Bot.* 91 1398–1408. 10.3732/ajb.91.9.1398 21652373

[B34] Harris N. (2006). The elevation history of the Tibetan Plateau and its implications for the Asian monsoon. *Palaeogeogr. Palaeoclimatol. Palaeoecol.* 241 4–15. 10.1016/j.palaeo.2006.07.009

[B35] He L. Jia K. H. Zhang R. G. Wang Y. Shi T. L. Li Z. C. (2021). Chromosome-scale assembly of the genome of Salix dunnii reveals a male-heterogametic sex determination system on chromosome 7. *Mol. Ecol. Resour.* 21 1966–1982. 10.1111/1755-0998.13362 33609314 PMC8359994

[B36] Hiroyoshi O. (2001). “Salicaceae of Japan,” in *The Science Reports of Tohoku University. 4th series Biology*, ed. Daigaku T. H. (Sendai: The Faculty of Science).

[B37] Hoang D. T. Chernomor O. von Haeseler A. Minh B. Q. Vinh L. S. (2018). UFBoot2: improving the ultrafast bootstrap approximation. *Mol. Biol. Evol.* 35 518–522. 10.1093/molbev/msx281 29077904 PMC5850222

[B38] Huang D. I. Cronk Q. C. (2015). Plann: a command-line application for annotating plastome sequences. *Appl. Plant Sci.* 3:1500026. 10.3732/apps.1500026 26312193 PMC4542940

[B39] Jiang D. Feng J. Dong M. Wu G. Mao K. Liu J. (2016). Genetic origin and composition of a natural hybrid poplar *Populus x jrtyschensis* from two distantly related species. *BMC Plant Biol.* 16:89. 10.1186/s12870-016-0776-6 27091174 PMC4836070

[B40] Kalyaanamoorthy S. Minh B. Q. Wong T. K. F. von Haeseler A. Jermiin L. S. (2017). ModelFinder: fast model selection for accurate phylogenetic estimates. *Nat. Methods* 14 587–589. 10.1038/nmeth.4285 28481363 PMC5453245

[B41] Katoh K. Kuma K. Toh H. Miyata T. (2005). MAFFT version 5: improvement in accuracy of multiple sequence alignment. *Nucleic Acids Res.* 33 511–518. 10.1093/nar/gki198 15661851 PMC548345

[B42] Kearse M. Moir R. Wilson A. Stones-Havas S. Cheung M. Sturrock S. (2012). Geneious Basic: an integrated and extendable desktop software platform for the organization and analysis of sequence data. *Bioinformatics* 28 1647–1649. 10.1093/bioinformatics/bts199 22543367 PMC3371832

[B43] Leaché A. D. Harris R. B. Rannala B. Yang Z. (2014). The influence of gene flow on species tree estimation: a simulation study. *Syst. Biol.* 63 17–30. 10.1093/sysbio/syt049 23945075

[B44] Li H. (2018). Minimap2: pairwise alignment for nucleotide sequences. *Bioinformatics* 34 3094–3100. 10.1093/bioinformatics/bty191 29750242 PMC6137996

[B45] Li H. Durbin R. (2009). Fast and accurate short read alignment with Burrows-Wheeler transform. *Bioinformatics* 25 1754–1760. 10.1093/bioinformatics/btp324 19451168 PMC2705234

[B46] Li H. Handsaker B. Wysoker A. Fennell T. Ruan J. Homer N. (2009). The sequence alignment/map format and SAMtools. *Bioinformatics* 25 2078–2079. 10.1093/bioinformatics/btp352 19505943 PMC2723002

[B47] Liang X.-Q. Ferguson D. K. Su T. Zhou Z.-K. (2016). Fossil leaves of *Populus* from the middle Miocene of Yunnan, SW China. *J. Syst. Evol.* 54 264–271. 10.1111/jse.12193

[B48] Liu X. Wang Z. Shao W. Ye Z. Zhang J. (2017). Phylogenetic and taxonomic status analyses of the *Abaso* section from multiple nuclear genes and plastid fragments reveal new insights into the North America origin of *Populus* (Salicaceae). *Front. Plant Sci.* 7:2022. 10.3389/fpls.2016.02022 28101098 PMC5209371

[B49] Liu Y. Li D. Zhang Q. Song C. Zhong C. Zhang X. (2017). Rapid radiations of both kiwifruit hybrid lineages and their parents shed light on a two-layer mode of species diversification. *New Phytol.* 215 877–890. 10.1111/nph.14607 28543189

[B50] Long C. Kubatko L. (2018). The effect of gene flow on coalescent-based species-tree inference. *Syst. Biol.* 67 770–785. 10.1093/sysbio/syy020 29566212

[B51] Malinsky M. Matschiner M. Svardal H. (2021). Dsuite - fast D-statistics and related admixture evidence from VCF files. *Mol. Ecol. Resour.* 21 584–595. 10.1111/1755-0998.13265 33012121 PMC7116594

[B52] Manchester S. R. Dilcher D. L. Tidwell W. D. (1986). Interconnected reproductive and vegetative remains of *Populus* (Salicaceae) from the Middle eocene Green River formation, Northeastern Utah. *Am. J. Bot.* 73 156–160. 10.1002/j.1537-2197.1986.tb09691.x 30139119

[B53] Manchester S. R. Judd W. S. Handley B. (2006). Foliage and fruits of early poplars (Salicaceae *Populus* from the Eocene of Utah, Colorado, and Wyoming. *Int. J. Plant Sci.* 167 897–908. 10.1086/503918

[B54] Matzke N. J. (2013). Probabilistic historical biogeography: new models for founder event speciation, imperfect detection, and fossils allow improved accuracy and model-testing. *Front. Biogeogr.* 5:242–248. 10.21425/F5FBG19694

[B55] McInerney F. A. Wing S. L. (2011). The Paleocene-Eocene thermal maximum: a perturbation of carbon cycle, climate, and biosphere with implications for the future. *Annu. Rev. Earth Planet. Sci.* 39 489–516. 10.1146/annurev-earth-040610-133431

[B56] McKenna A. Hanna M. Banks E. Sivachenko A. Cibulskis K. Kernytsky A. (2010). The genome analysis toolkit: a mapreduce framework for analyzing next-generation DNA sequencing data. *Genome Res.* 20 1297–1303. 10.1101/gr.107524.110 20644199 PMC2928508

[B57] Milne R. I. Abbott R. J. (2002). The origin and evolution of tertiary relict floras. *Adv. Bot. Res.* 38 281–314.

[B58] Mona H. Pierre P. Selvadurai D. (2006). Genetic relationships among species of *Populus* (Salicaceae) based on nuclear genomic data. *J. Torrey Bot. Soc.* 133 519–527.

[B59] Nazarov M. I. (1936). “Family XI. Salicaceae line,” in *Flora of The USSR*, ed. Komarove A. V. L. (Aptekarsky: Botanical Institute of the Academy of Sciences of the U.S.S.R).

[B60] Nguyen L.-T. Schmidt H. A. von Haeseler A. Minh B. Q. (2014). IQ-TREE: a fast and effective stochastic algorithm for estimating maximum-likelihood phylogenies. *Mol. Biol. Evol.* 32 268–274. 10.1093/molbev/msu300 25371430 PMC4271533

[B61] Passchier S. Bohaty S. M. Jiménez-Espejo F. Pross J. Röhl U. van de Flierdt T. (2013). Early eocene to middle Miocene cooling and aridification of East Antarctica. *Geochem. Geophys. Geosyst.* 14 1399–1410. 10.1002/ggge.20106

[B62] Pelser P. B. Kennedy A. H. Tepe E. J. Shidler J. B. Nordenstam B. Kadereit J. W. (2010). Patterns and causes of incongruence between plastid and nuclear Senecioneae (Asteraceae) phylogenies. *Am. J. Bot.* 97 856–873. 10.3732/ajb.0900287 21622451

[B63] Purcell S. Neale B. Todd-Brown K. Thomas L. Ferreira M. A. R. Bender D. (2007). PLINK: a tool set for whole-genome association and population-based linkage analyses. *Am. J. Hum. Genet.* 81 559–575. 10.1086/519795 17701901 PMC1950838

[B64] Qiu Y.-X. Fu C.-X. Comes H. P. (2011). Plant molecular phylogeography in China and adjacent regions: tracing the genetic imprints of Quaternary climate and environmental change in the world’s most diverse temperate flora. *Mol. Phylogenet. Evol.* 59 225–244. 10.1016/j.ympev.2011.01.012 21292014

[B65] Rieseberg L. H. Soltis D. E. (1991). Phylogenetic consequences of cytoplasmic gene flow in plants. *Evol. Trends Plants* 5 65–84.

[B66] Riesselman C. R. Dunbar R. B. Mucciarone D. A. Kitasei S. S. (2007). *High Resolution Stable Isotope and Carbonate Variability During the Early Oligocene Climate Transition: Walvis Ridge (ODP Site 1263). in Open-File Report.* Reston, VA: United States Geological Survey.

[B67] Schneider C. K. (1916). “Salicaceae, *Populus*,” in *Plantae Wilsonianae*, ed. Sargent C. S. (Cambridge: Cambridge University Press).

[B68] Schroeder H. Fladung M. (2010). SSR and SNP markers for the identification of clones, hybrids and species within the genus *Populus*. *Silvae Genet.* 59 257–263. 10.1515/sg-2010-0036

[B69] Shen X. Ren Y. Ma X. Feng X. Zhang S. Wang G. (2018). Clustering analysis and biogeographical regionalization of distribution patterns of dicotyledonous plants in the world — biogeographical regionalization research X (in Chinese). *Bot. Res.* 7 405–417. 10.12677/br.2018.74049

[B70] Skvortsov A. K. (2009). The genus *Populus* L. (Salicaceae) of Indian Himalaya. *Novosti Sist. Vyssh. Rast.* 40 52–67.

[B71] Slavov G. T. Zhelev P. (2012). “Salient biological features, systematics, and genetic variation of *Populus*,” in *Genetics and Genomics of Populus*, eds Jansson S. Bhalerao R. Groover A. (Berlin: Springer).

[B72] Smith R. L. Sytsma K. J. (1990). Evolution of *Populus nigra* (Sect. *Aigeiros*): introgressive hybridization and the chloroplast contribution of *Populus alba* (Sect. *Populus*). *Am. J. Bot.* 77 1176–1187. 10.1002/j.1537-2197.1990.tb13616.x

[B73] Solís-Lemus C. Ané C. (2016). Inferring phylogenetic networks with maximum pseudolikelihood under incomplete lineage sorting. *PLoS Genet.* 12:e1005896. 10.1371/journal.pgen.1005896 26950302 PMC4780787

[B74] Stamatakis A. (2014). RAxML version 8: a tool for phylogenetic analysis and post-analysis of large phylogenies. *Bioinformatics* 30 1312–1313. 10.1093/bioinformatics/btu033 24451623 PMC3998144

[B75] Stettler R. Bradshaw H. Heilman P. E. Hinckley T. (1996). *Biology of Populus.* Ottawa, ON: NRC Research Press.

[B76] Su T. Spicer R. A. Li S.-H. Xu H. Huang J. Sherlock S. (2019). Uplift, climate and biotic changes at the Eocene–Oligocene transition in south-eastern Tibet. *Natl. Sci. Rev.* 6 495–504. 10.1093/nsr/nwy062 34691898 PMC8291530

[B77] Sun T. (1986). Two new species and six variaties of *Populus* from Inner-mongolica (in Chinese). *J. Nanjing For. Univ.* 4 109–117.

[B78] Swofford D. L. (2003). *PAUP*. Phylogenetic Analysis Using Parsimony (* and Other Methods).* Sunderland, MA: Sinauer Associates.

[B79] Takuno S. Kado T. Sugino R. P. Nakhleh L. Innan H. (2012). Population genomics in bacteria: a case study of *Staphylococcus aureus*. *Mol. Biol. Evol.* 29 797–809. 10.1093/molbev/msr249 22009061 PMC3350317

[B80] Tiffney B. (2000). Geographic and climatic influence on the Cretaceous and Tertiary history of Euramerican floristic similarity. *Acta. Univ. Carol. Geol.* 44 5–16.

[B81] Tiffney B. H. Manchester S. R. (2001). The use of geological and paleontological evidence in evaluating plant phylogeographic hypotheses in the northern Hemisphere Tertiary. *Int. J. Plant Sci.* 162 S3–S17. 10.1086/323880

[B82] Tsitrone A. Kirkpatrick M. Levin D. A. (2003). A model for chloroplast capture. *Evolution* 57 1776–1782. 10.1111/j.0014-3820.2003.tb00585.x 14503619

[B83] Tuskan G. A. Difazio S. Jansson S. Bohlmann J. Grigoriev I. Hellsten U. (2006). The genome of black cottonwood, *Populus trichocarpa* (Torr. & Gray). *Science* 313 1596–1604. 10.1126/science.1128691 16973872

[B84] Wang C. Tung S.-L. (1982). New taxa of *Populus* (II) (in Chinese). *Bull. Bot. Res.* 2 105–120.

[B85] Wang M. Zhang L. Zhang Z. Li M. Wang D. Zhang X. (2020). Phylogenomics of the genus *Populus* reveals extensive interspecific gene flow and balancing selection. *New Phytol.* 225 1370–1382. 10.1111/nph.16215 31550399

[B86] Wang Z. Du S. Dayanandan S. Wang D. Zeng Y. Zhang J. (2014). Phylogeny reconstruction and hybrid analysis of *Populus* (Salicaceae) based on nucleotide sequences of multiple single-copy nuclear genes and plastid fragments. *PLoS One* 9:e103645. 10.1371/journal.pone.0103645 25116432 PMC4130529

[B87] Wolfe J. A. (1980). Tertiary climates and floristic relationships at high latitudes in the northern hemisphere. *Palaeogeogr. Palaeoclimatol. Palaeoecol.* 30 313–323. 10.1016/0031-0182(80)90063-2

[B88] Wu C. I. (1991). Inferences of species phylogeny in relation to segregation of ancient polymorphisms. *Genetics* 127:429. 10.1093/genetics/127.2.429 2004713 PMC1204370

[B89] Xu P. Zhang X. Wang X. Li J. Liu G. Kuang Y. (2014). Genome sequence and genetic diversity of the common carp, *Cyprinus carpio*. *Nat. Genet.* 46:1212. 10.1038/ng.3098 25240282

[B90] Xu X. Liu X. Ge S. Jensen J. D. Hu F. Li X. (2012). Resequencing 50 accessions of cultivated and wild rice yields markers for identifying agronomically important genes. *Nat. Biotechnol.* 30 105–111. 10.1038/nbt.2050 22158310

[B91] Yang Z. (2007). PAML 4: phylogenetic analysis by maximum likelihood. *Mol. Biol. Evol.* 24 1586–1591. 10.1093/molbev/msm088 17483113

[B92] Yu Y. Harris A. J. Blair C. He X. (2015). RASP (reconstruct ancestral state in phylogenies): a tool for historical biogeography. *Mol. Phylogenet. Evol.* 87 46–49. 10.1016/j.ympev.2015.03.008 25819445

[B93] Yun T. Li J. M. Zhou A. P. Yan L. X. Zong D. Li D. (2015). Analysis of phylogenetic relationship of *Populus* based on sequence data of chloroplast regions (in Chinese). *Plant Physiol.* 51 1339–1346. 10.13592/j.cnki.ppj.2015.0118

[B94] Zachos J. C. Kump L. R. (2005). Carbon cycle feedbacks and the initiation of Antarctic glaciation in the earliest Oligocene. *Glob. Planet. Change* 47 51–66. 10.1016/j.gloplacha.2005.01.001

[B95] Zachos J. Pagani M. Sloan L. Thomas E. Billups K. (2001). Trends, rhythms, and aberrations in global climate 65 Ma to present. *Science* 292 686–693. 10.1126/science.1059412 11326091

[B96] Zhang C. Rabiee M. Sayyari E. Mirarab S. (2018). ASTRAL-III: polynomial time species tree reconstruction from partially resolved gene trees. *BMC Bioinformatics* 19:153. 10.1186/s12859-018-2129-y 29745866 PMC5998893

[B97] Zhang L. Wang M. Guo X. Ma T. (2018). Plastome phylogeny and lineage diversification of Salicaceae with focus on poplars and willows. *Ecol. Evol.* 8 7817–7823. 10.1002/ece3.4261 30250665 PMC6145263

[B98] Zhang W. Dasmahapatra K. K. Mallet J. Moreira G. R. Kronforst M. R. (2016). Genome-wide introgression among distantly related Heliconius butterfly species. *Genome Biol.* 17:25. 10.1186/s13059-016-0889-0 26921238 PMC4769579

[B99] Zhao N. Liu J. (2001). The family Salicaceae in Tibet (in Chinese). *J. Sichuan For. Sci. Technol.* 22 1–18. 10.1186/1471-2164-12-465 21943393 PMC3188535

[B100] Zhou X. Wang B. Pan Q. Zhang J. Kumar S. Sun X. (2014). Whole-genome sequencing of the snub-nosed monkey provides insights into folivory and evolutionary history. *Nat. Genet.* 46 1303–1310. 10.1038/ng.3137 25362486

[B101] Zong D. Gan P. Zhou A. Zhang Y. Zou X. Duan A. (2019). Plastome sequences help to resolve deep-level relationships of *Populus* in the family Salicaceae. *Front. Plant Sci.* 10:5. 10.3389/fpls.2019.00005 30723484 PMC6349946

[B102] Zsuffa L. (1975). “A summary review of interspecific breeding in the genus *Populus*,” in *Proceedings of the 14th Annual Meeting of the Canadian Tree Improvement Association* (Ottawa, ON: Canadian Forest Service).

