# Supplementary material for: Phylogenomics and Biogeography of Populus Based on Comprehensive Sampling Reveal Deep-Level Relationships and Multiple Intercontinental Dispersals
Source: Front Plant Sci. 2022 Feb 4;13:813177. doi: 10.3389/fpls.2022.813177 (PMC8855119; doi:10.3389/fpls.2022.813177)
Supplement: Supplementary file 2 [file Data_Sheet_1.docx]

Supplementary Material

**Supplementary Figure 1.** Phylogeny inferred from SVDquartets based on all nuclear SNPs. Unless otherwise indicated, all nodes had 100% supports of bootstrap.

**Supplementary Figure 2.** Phylogenetic relationship of 29 *Populus* taxa (80 samples) reconstructed by IQ-TREE based on 12.93 million nuclear SNPs from Wang et al. (2020). Unless otherwise indicated, all nodes had 100% supports of SH-aLRT bootstrap (Alrt) and Ultrafast bootstrap (UFBoot).

**Supplementary Figure 3.** Phylogeny with the branch length of genus *Populus* reconstructed by IQ-TREE based on complete chloroplast genomes. Ultrafast bootstrap support (UFBoot), and SH-aLRT bootstrap support (Alrt) are not shown.

**Supplementary Figure 4.** Plots of the first two principal components for SNP data from 103 individuals from six sections of the genus *Populus*.

**Supplementary Figure 5.** *D*-statistics from ABBA-BABA tests shows extensive gene flow of *Populus*. (a) Sect. *Abaso* is closer to subgen. *Eupopulus* than any other sections; (b) *P. nigra* is closer to P. alba than any other species of sect. *Populus*; (c) *D*-statistics between *P. pseudomaximowiczii* and other species of subgen. *Eupopulus*, *D*-statistics with Z-score < 3 are not shown, which indicated statistically insignificant. (d) *D*-statistics between *P. szechuanica* var. *tivetica* and other species of subgen. *Eupopulus*, *D*-statistics with *Z*-score < 3 are not shown.

**Supplementary Table 1.** Summary of the statistics of genome resequencing data for 103 individuals of 54 species and two outgroups. The individuals for which genome sequences were downloaded from the Genome Sequence Archive (GSA) are marked by asterisks, while those downloaded from the NCBI are marked by the pound sign.

**Supplementary Table 2.** Assemble and annotation information of the plastomes of *Populus* and two outgroups.

**Supplementary Table 3.** The functional annotation statistics of SNP in *Populus* annotated by SnpEff.

**Supplementary Table 4.** Summaries of the five subclades of sect. *Aigeiros*-*Tacamahaca* clade in the nuclear SNPs tree. The numbers in brackets indicate the number of variety or hybrid.

**Supplementary Table 5.** Detail information of *D*-statistics tests for all comparisons. Only *D*-statistics greater than zero were calculated and recorded.

# Reference

Wang M., Zhang L., Zhang Z., Li M., Wang D., Zhang X.*, et al.* (2020). Phylogenomics of the genus *Populus* reveals extensive interspecific gene flow and balancing selection. *New Phytol*. doi: 10.1111/nph.16215.
