# Supplementary figures and images for: Phylogenomics and Biogeography of Populus Based on Comprehensive Sampling Reveal Deep-Level Relationships and Multiple Intercontinental Dispersals
Source: Front Plant Sci. 2022 Feb 4;13:813177. doi: 10.3389/fpls.2022.813177 (PMC8855119; doi:10.3389/fpls.2022.813177)

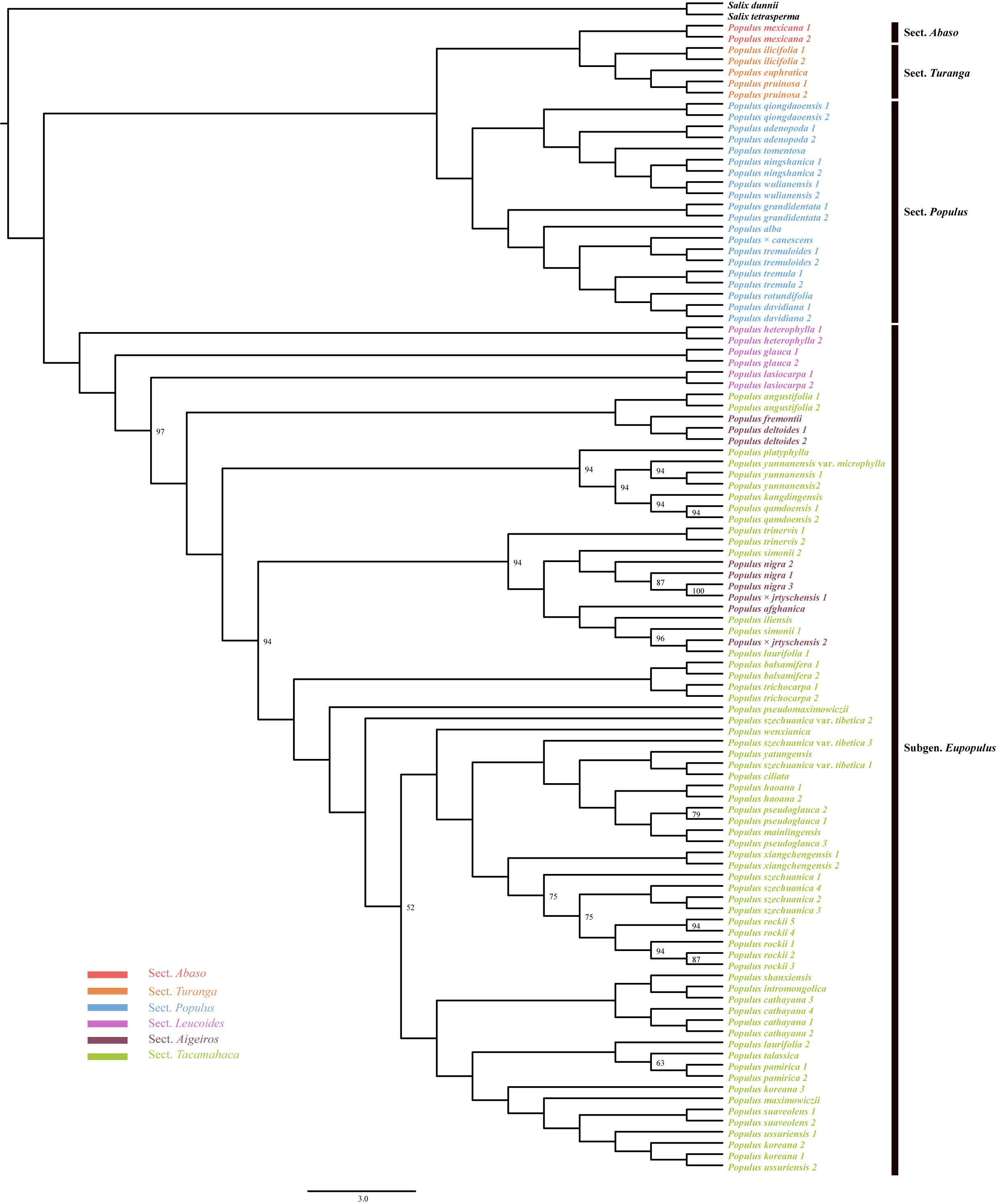

Supplement: Supplementary file 3 [file Image_1.JPEG]

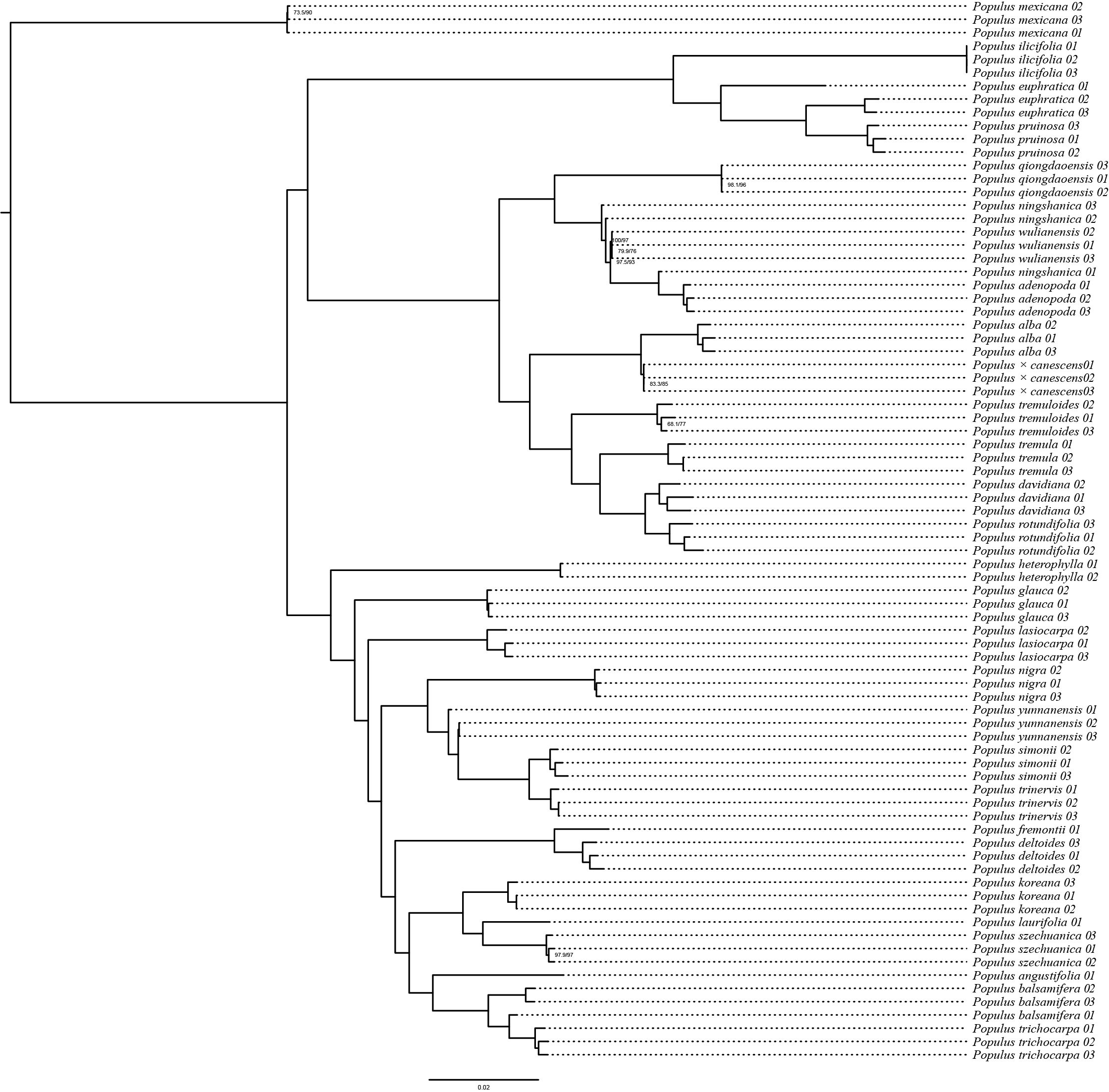

Supplement: Supplementary file 4 [file Image_2.JPEG]

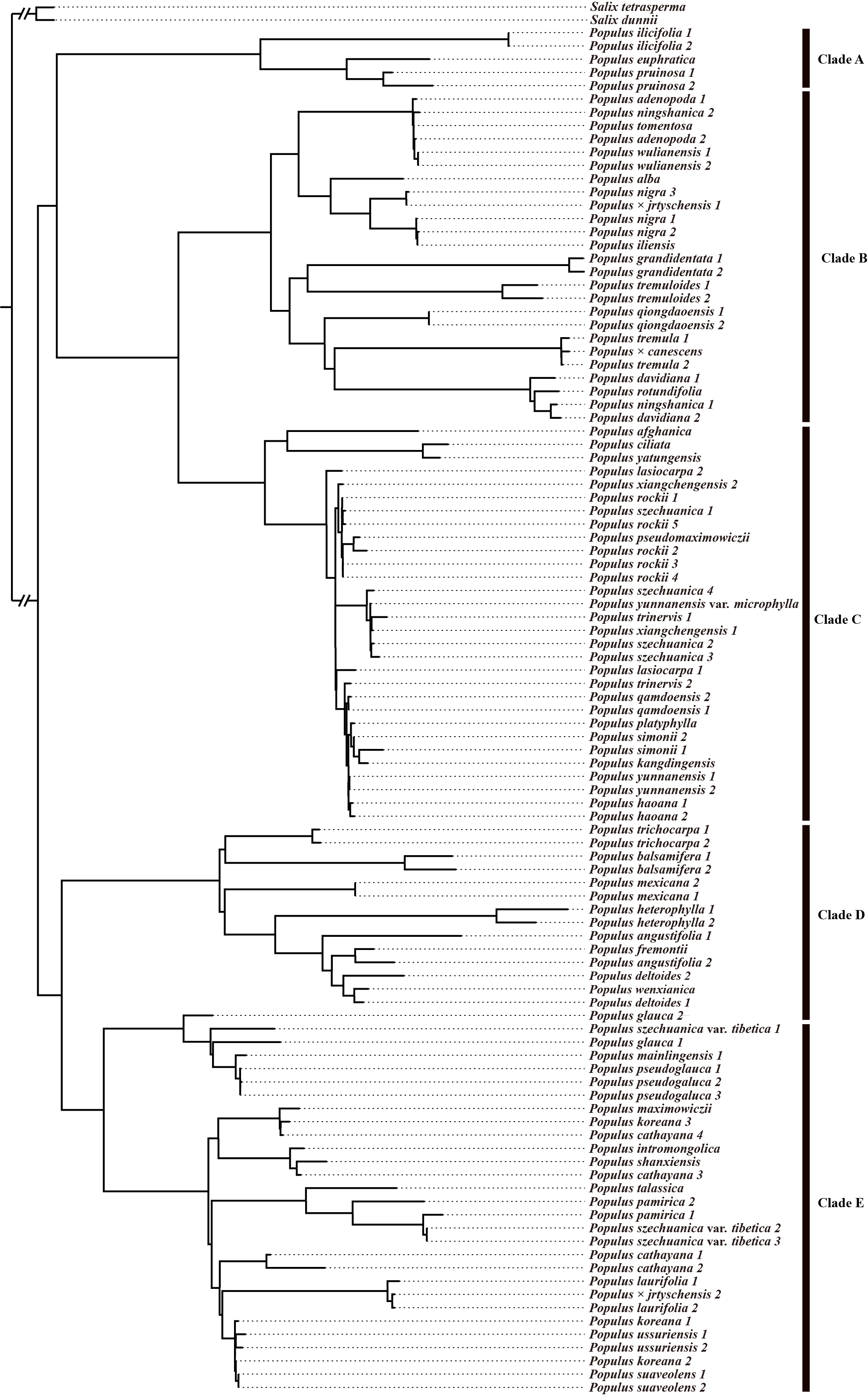

Supplement: Supplementary file 5 [file Image_3.JPEG]

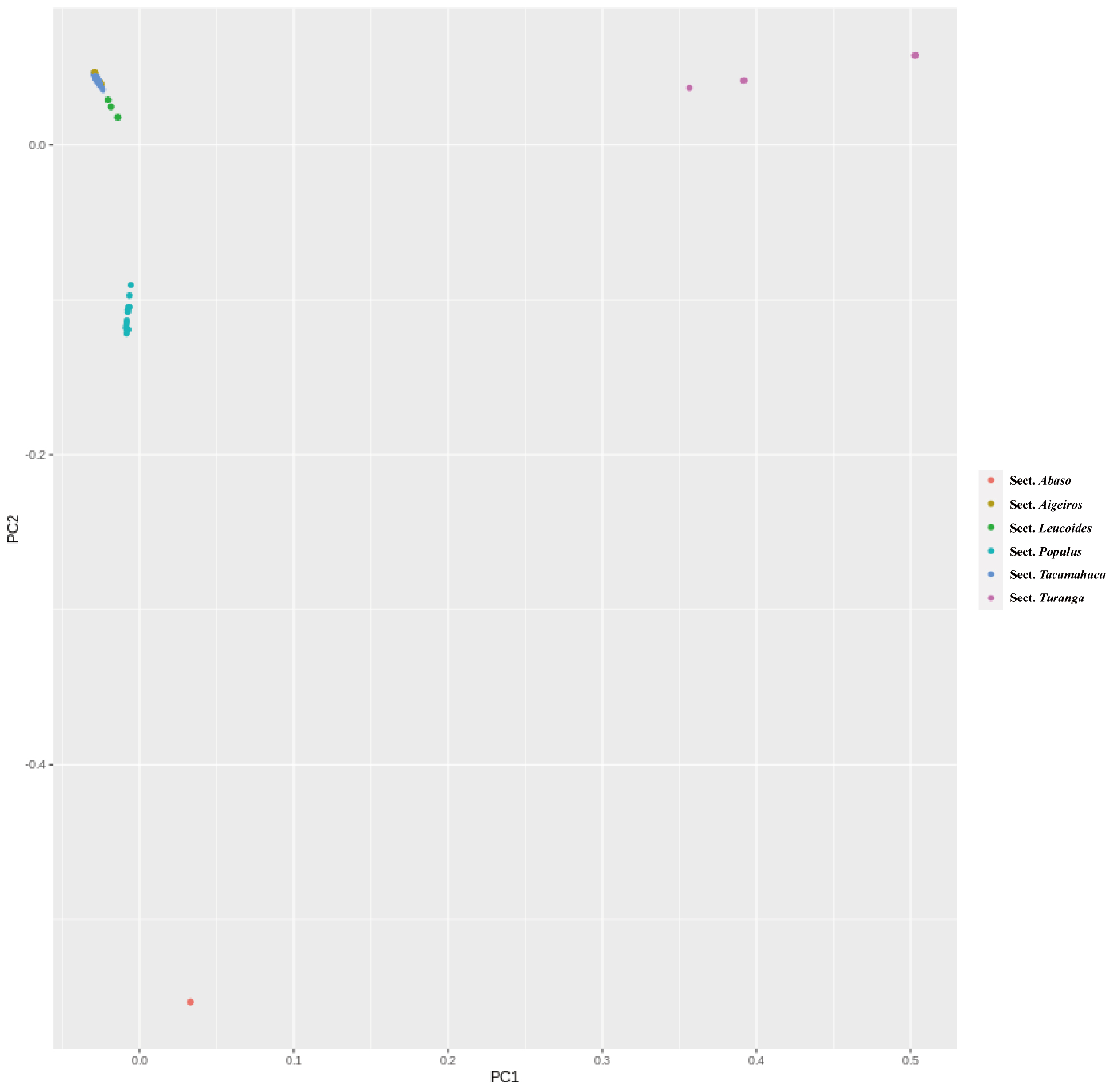

Supplement: Supplementary file 6 [file Image_4.JPEG]

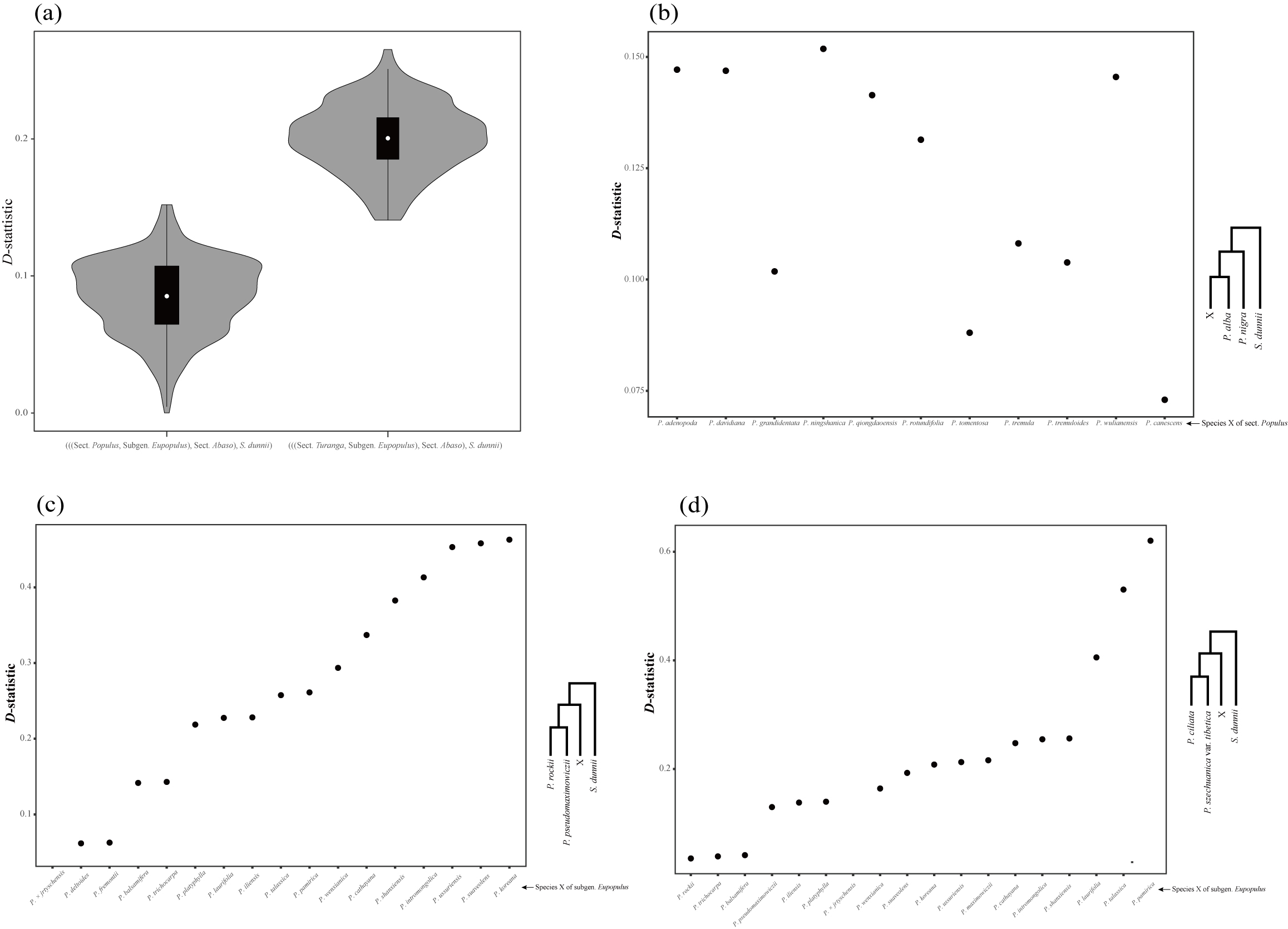

Supplement: Supplementary file 7 [file Image_5.JPEG]
